# Neurophysiological Correlates of Attentional Fluctuation in Attention-Deficit/Hyperactivity Disorder

**DOI:** 10.1007/s10548-017-0554-2

**Published:** 2017-03-14

**Authors:** Celeste H. M. Cheung, Gráinne McLoughlin, Daniel Brandeis, Tobias Banaschewski, Philip Asherson, Jonna Kuntsi

**Affiliations:** 1 MRC Social, Genetic and Developmental Psychiatry Centre, Institute of Psychiatry, Psychology and Neuroscience, King’s College London, London, UK; 2 Department of Child and Adolescent Psychiatry and Psychotherapy, Central Institute of Mental Health, Medical Faculty Mannheim, Heidelberg University, Mannheim, Germany; 3 Department of Child and Adolescent Psychiatry, University of Zurich, Zurich, Switzerland; 4 Center for Integrative Human Physiology, University of Zurich, Zurich, Switzerland; 5 Neuroscience Center Zurich, University of Zurich, Zurich, Switzerland

**Keywords:** ADHD, P3, Reaction time variability, CNV, ERP, EEG

## Abstract

Cognitive performance in attention-deficit/hyperactivity disorder (ADHD) is characterised, in part, by frequent fluctuations in response speed, resulting in high reaction time variability (RTV). RTV captures a large proportion of the genetic risk in ADHD but, importantly, is malleable, improving significantly in a fast-paced, rewarded task condition. Using the temporal precision offered by event-related potentials (ERPs), we aimed to examine the neurophysiological measures of attention allocation (P3 amplitudes) and preparation (contingent negative variation, CNV), and their associations with the fluctuating RT performance and its improvement in ADHD. 93 participants with ADHD and 174 controls completed the baseline and fast-incentive conditions of a four-choice reaction time task, while EEG was simultaneously recorded. Compared to controls, individuals with ADHD showed both increased RTV and reduced P3 amplitudes during performance on the RT task. In the participants with ADHD, attenuated P3 amplitudes were significantly associated with high RTV, and the increase in P3 amplitudes from a slow baseline to a fast-paced, rewarded condition was significantly associated with the RTV decrease. Yet, the individuals with ADHD did not show the same increase in CNV from baseline to fast-incentive condition as observed in controls. ADHD is associated both with a neurophysiological impairment of attention allocation (P3 amplitudes) and an inability to adjust the preparatory state (CNV) in a changed context. Our findings suggest that both neurophysiological and cognitive performance measures of attention are malleable in ADHD, which are potential targets for non-pharmacological interventions.

**Electronic supplementary material:**

The online version of this article (doi:10.1007/s10548-017-0554-2) contains supplementary material, which is available to authorized users.

## Introduction

Inconsistent performance on reaction time tasks is one of the most prominent features of cognitive performance in ADHD (Kofler et al. 2013; Tamm et al. 2012). Frequent fluctuations in response speed result in high reaction time variability (RTV), which is one of the most investigated cognitive performance deficits in ADHD research over the past decade (Castellanos et al. 2005; Kofler et al. 2013; Kuntsi and Klein 2012; Tamm et al. 2012). Less well investigated, but potentially clinically more promising, is the observation that individuals with ADHD show a significantly greater reduction in RTV under a rewarded task condition compared to individuals without ADHD (Kuntsi et al. 2012). Identifying the neurophysiological basis of such improvement could inform the development of brain training programs for ADHD that focus on reaching and maintaining an optimal state of alertness.

Inducing an optimal state of alertness is challenging, as the effectiveness of task manipulations likely depends on both individual and task factors, such as the age of participants and the length and nature of the overall test battery. Yet several studies have succeeded in demonstrating an ADHD-sensitive improvement in RTV following the introduction of rewards (with or without an additional manipulation with a faster event rate) (Andreou et al. 2007; Slusarek et al. 2001; Uebel et al. 2010). While studies that have examined separately the effects of rewards and a faster event rate within the same sample are suggestive of rewards leading to a greater improvement in RTV (Banaschewski et al. 2012; Kuntsi et al. 2012; Uebel et al. 2010), we recently demonstrated, using genetic model fitting across two large sibling and twin samples, that 74–83% of the underlying aetiology is shared between RTV improvement following rewards and a faster event rate (Kuntsi et al. 2012). We further demonstrated that RTV baseline performance (in a slow-unrewarded condition) measures the same aetiological process as captured by the RTV improvement across conditions (from the baseline condition to a fast rewarded condition) (Kuntsi et al. 2012). These findings support theories that emphasise the malleability of the observed high RTV in ADHD, such as those that link ADHD to difficulties regulating arousal (Halperin et al. 2008; Johnson et al. 2007; O’Connell et al. 2009; Sergeant 2005; Van der Meere 2002). While RTV captures a large proportion of the familial influences underlying ADHD, it largely separates from a second familial cognitive impairment in ADHD that captures executive control processes, such as response inhibition (Kuntsi et al. 2010).

In addition to twin and family studies, the underpinnings of RTV have also been examined in initial neuroimaging and neurophysiological studies. Functional magnetic resonance imaging (fMRI) studies have reported that ADHD and healthy controls show differential patterns of brain activation related to RTV. In adolescents with ADHD, increased RTV was associated with decreased basal ganglia and thalamus activity (Rubia et al. 2007), increased pre-supplementary motor and decreased prefrontal activity (Suskauer et al. 2008). In contrast, in controls increased RTV was associated with increased temporal lobe activation (Rubia et al. 2007), reduced pre-supplementary motor and increased prefrontal activity (Suskauer et al. 2008). In a recent EEG oscillatory twin study the theta phase variability from frontal midline cortex emerged as a neurophysiological marker that was significantly associated with RTV and ADHD both phenotypically and genetically (McLoughlin et al. 2014).

At the level of event-related potentials (ERP), RTV has previously been linked to the P3 component in a neurotypical population (Saville et al. 2011) and to the slow cortical potential contingent negative variation (CNV) in a clinical population with ADHD (Kratz et al. 2012). The P3 wave is time-locked to cognitive aspects of a stimulus appearing between 250 and 450 ms following stimulus presentation (Sutton et al. 1965) and typically identified as a parieto-central positive deflection. P3 latency has been shown to be associated with mean RT (Nieuwenhuis et al. 2005; Verleger 1997), but studies that examined RTV have found this variable to relate only to the amplitude rather than the latency of the P3 component (Ramchurn et al. 2014; Segalowitz et al. 1997). P3 amplitudes have been considered to be associated with the amount of attention allocated to a task (Donchin et al. 1986; Kok 1990). This hypothesis stems from findings in dual-task studies indicating a significant positive relationship between P3 amplitude and task demands (Kok 1990; Polich 2007). Additional evidence supporting the role of P3 in attentional processing is provided by studies that showed a significant reduction in P3 amplitudes when participant’s attention was directed away from the task (Johnson 1988; Mangun and Hillyard 1990), and larger P3 amplitudes to attended than unattended target stimuli (Kok 1990). On the other hand, recent studies have also identified a posterior P3 reflecting neural decision-making processes that are time-locked to response execution (Kelly and O’Connell 2013; Twomey et al. 2015). Investigating the response-aligned neural signal in addition to the target-aligned ERP in the P3 range should thus help to disentangle the potential sources of increased RTV. P3 amplitudes are attenuated in children and adults with ADHD (Szuromi et al. 2011; Tye et al. 2011). While most studies focus on target-related attenuations of the P3 in ADHD, similar (Steger et al. 2000), or even stronger attenuations of response-related P3 activity (Saville et al. 2015) have also been reported. While there is some evidence for normalisation in P3 amplitudes in ADHD following stimulant medication (Overtoom et al. 2009; Pliszka 2007), limited research has investigated whether P3 amplitudes can be altered using non-pharmacological techniques. Initial findings from both children and adults with ADHD using a go/no-go task revealed a greater-than-expected increase in P3 amplitude from a slow to a faster condition (Wiersema et al. 2006a, b), indicated by a significant group by condition interaction. Incentives based on number of points earned also enhanced P3 amplitudes, but similarly in participants with and without ADHD (Groom et al. 2010).

The CNV is a slow negative potential that occurs after a warning stimulus in anticipation of the target stimulus. This preparatory ERP component reflects cognitive anticipation and motor preparation (Albrecht et al. 2013; Bender et al. 2005; Segalowitz et al. 1997), undergoing prominent late maturation from a parietal to a fronto-central negative topography (Bender et al. 2005, Doehnert et al. 2013). Reduced CNV in ADHD is a consistent finding (Albrecht et al. 2013; Banaschewski et al. 2003; McLoughlin et al. 2010), and has been considered as a candidate endophenotype that shows strong familial influences with ADHD (Albrecht et al. 2013; Rommelse et al. 2007). Methylphenidate (MPH) was found to simultaneously reduce RTV and increase CNV in ADHD (Kratz et al. 2012), with a significant correlation found between the two measures (r = 0.34). Several studies that assessed the effectiveness of neurofeedback training on slow cortical potentials in ADHD indicated an increase in CNV amplitudes, which was associated with a reduction in ADHD symptomatology (Heinrich et al. 2004; Wangler et al. 2011), or less CNV habituation with better training performance (Doehnert et al. 2008). These findings allude to the malleability of the CNV and its relationship with RTV. The CNV is typically calculated as the mean amplitude at Cz or Pz, averaged over a few hundred milliseconds before target onset (Albrecht et al. 2013; Banaschewski et al. 2003; Segalowitz et al. 1997), although the site or region and interval varies across studies depending on the age group and paradigm design.

Only one study to date has examined the relationship between P3 and RTV in relation to the preparatory CNV (Segalowitz et al. 1997). This study examined patients with traumatic brain injury (TBI), who had impaired attentional processing as reflected by increased RTV, and found a strong negative association between RTV and P3 amplitudes. In addition, significant relationships between RTV and the CNV, and between P3 and the CNV, were also observed (Segalowitz et al. 1997).

To investigate the neural basis of attentional variability in ADHD using an RT task with strong phenotypic and genetic association with ADHD and demonstrated ADHD-sensitive improvement across conditions (Andreou et al. 2007; Banaschewski et al. 2012; Kuntsi et al. 2012, 2009), we focus on a parietal P3 amplitudes and the preparatory CNV across baseline (slow-unrewarded) and fast-incentive conditions of the Fast Task in a large sample of ADHD and control participants. The Fast Task (Kuntsi et al. 2005) is a four-choice RT task that compares a slow and unrewarded condition performance with performance in a fast-incentive condition that specifically rewards a reduction in RTV (unlike go/no-go tasks that reward inhibition performance).

We aimed, first, to establish, using a large follow-up sample, whether ADHD continues to be associated with a greater-than-expected RTV improvement across the task conditions in adolescence and early adulthood. Second, we aimed to investigate whether a similar pattern (greatest impairment in participants with ADHD in the baseline condition and a greater improvement between conditions in participants with ADHD than controls) is observed also for the attentional P3 and the CNV. Third, we aimed to examine the relationship between RTV, P3 and CNV within and across task conditions. As lower IQ is associated with ADHD (Kuntsi et al. 2004; Wood et al. 2011), we systematically examined the effects of IQ by running our analyses with and without IQ as a covariate.

## Materials and Methods

### Sample

ADHD and control participants who had taken part in our previous research (Chen et al. 2008; Kuntsi et al. 2010) were invited to take part in this study. ADHD participants were included if they had ADHD in childhood and met DSM-IV criteria for any ADHD subtype at follow up. Exclusion criteria included IQ < 70, autism, epilepsy, general learning difficulties, brain disorders and any genetic or medical disorder associated with externalising behaviours that might mimic ADHD. The authors assert that all procedures contributing to this work comply with the ethical standards of the relevant national and institutional committees on human experimentation and with the Helsinki Declaration of 1975, as revised in 2008.

Seven ADHD participants were excluded from the analyses: two became very drowsy and could not complete the task, in two cases there was EEG equipment failure, and in three cases there were less than 20 acceptable segments available as required for averaging of EEG data. Two control participants were excluded, as they met ADHD criteria based on parent report. The final follow-up sample consisted of 93 ADHD participants (8 sibling pairs and 77 singletons; mean age = 18.28, SD = 2.98) and 174 controls (81 sibling pairs and 12 singletons; mean age = 17.76, SD = 2.16). The two groups did not differ in age (t = 1.56, df = 178, p = 0.16) or gender (χ^2^ = 1.38, df = 1, p = 0.24), but a significant difference in IQ was observed (t = −6.85, df = 178, p < 0.01) (Table S1 and Figure S2).

### Procedure

The Fast Task was administered as part of a longer assessment session at the research centre. A 48-hour ADHD medication-free period was required. Face-to-face or telephone clinical interviews were administered to the parent of each ADHD proband shortly before or after the participant’s assessment.

### Measures

#### ADHD Diagnosis

The Diagnostic Interview for ADHD in Adults [DIVA, (Kooij and Francken 2007)], a semi-structured interview based on the DSM-IV criteria, was conducted with the ADHD proband and the parent separately for current symptoms only, because in all cases a clinical and research diagnosis of combined type ADHD had already been established (Chen et al. 2008). The Barkley’s functional impairment scale [BFIS; (Barkley and Murphy 2006)] was used to assess functional impairments commonly associated with ADHD in five areas of their everyday life. Each item ranges from 0 (never or rarely) to 3 (very often). Participants were classified as “affected”, if they scored a “yes” on ≥6 items on the DIVA for either inattention or hyperactivity-impulsivity based on parent report, and scored ≥2 on ≥2 areas of impairments on the BFIS, rated by their parent.

#### IQ

The vocabulary and block design subtests of the Wechsler Abbreviated Scale of Intelligence (WASI) (Wechsler 1999) were administered to all participants to derive an estimate of IQ.

#### The Fast Task (Andreou et al. 2007; Kuntsi et al. 2006)

The baseline (slow-unrewarded) condition followed a standard warned four-choice RT task. A warning signal (four empty circles, arranged side by side) first appeared on the screen. At the end of the fore-period (presentation interval for the warning signal), the circle designated as the target signal for that trial was filled (coloured) in. The participant was asked to make a compatible choice by pressing the response key that directly corresponded in position to the location of the target stimulus. Following a response, the stimuli disappeared from the screen and a fixed inter-trial interval of 2.5 s followed. Speed and accuracy were emphasised equally. If the participant did not respond within 10 s, the trial terminated. First, a practice session was administered, during which the participant had to respond correctly to five consecutive trials. The baseline condition, with a fore-period of 8 s and consisting of 72 trials, then followed.

To investigate the extent to which a response style characterised by slow and variable speed of responding can be maximally reduced, the task includes a comparison condition that uses a fast event rate (fore-period of 1 s) and incentives. This condition started immediately after the baseline condition and consisted of 80 trials, with a fixed inter-trial interval of 2.5 s following the response. If the participant did not respond within 10 s, the trial terminated. Speed and accuracy were emphasised equally. The participants were told to respond really quickly one after another, to win smiley faces and earn real prizes in the end. Participants won a smiley face for responding faster than their own MRT during the baseline (first) condition consecutively for three trials. The smiley faces are continuously updated and remain on the screen throughout this condition until the end of the task. The baseline MRT was calculated here based on the middle 94% of responses (the exclusion of the top and bottom 3% of responses is only used when calculating a baseline mean RT for the set-up of the fast-incentive condition, and is not used for analyses), therefore excluding extremely fast and extremely slow responses. The smiley faces appeared below the circles in the middle of the screen and were updated continuously. The fast-incentive condition is always administered after the baseline condition and, as such, does not involve a similar learning phase. Participants earned £5 in cash after the task battery.

Due to the longer fore-period in the baseline condition, the two conditions were not matched on task length, but were matched on the number of trials. We analysed RTV performance, defined as within-subject standard deviation of mean RT on all correct trials, in both the full baseline condition and separately on the length-matched segment (Andreou et al. 2007). However, we did not control for task length in the ERP analyses, as data from the full baseline condition was required to obtain adequate trials for ERP averaging.

### EEG Recording and Analysis

The EEG was recorded from 62 channels DC-coupled recording system (extended 10–20 montage), with a 500 Hz sampling-rate, impedances kept under 10kΩ, and FCz as the recording reference. The electro-oculograms (EOGs) were recorded from two additional electrodes above and below the left eye and at the outer canthi.

The EEG data were analysed using Brain Vision Analyzer (2.0) (Brain Products, Germany). After down-sampling the data to 256 Hz, the EEG data were re-referenced to the average and filtered offline with digital band-pass (0.1–30 Hz, 24 dB/oct) Butterworth filters. Ocular artifacts were identified from the data using Independent Component Analysis [ICA, (Jung et al. 2000)]. The extracted independent components were manually inspected and ocular artefacts were removed by back-projection of all but those components. All ERP averages contained at least 20 accepted sweeps. Data with other artifacts exceeding ±100 μV in any channel were rejected. P3 amplitude was analysed as the area amplitude measure (μV*ms) at Pz between 250 and 450 ms following the target, to reduce bias due to the varying noise levels induced by the different task conditions (Luck 2005). For the main P3 analyses, all the accepted trials were baseline-corrected by subtracting the mean activity (200 ms prior to the stimulus target onset) from the P3 ERPs. The mean amplitudes of this pre-target period [−200–0 ms, using a technical zero baseline (Albrecht et al. 2013; Banaschewski et al. 2003)] at Cz and Pz were also analysed separately as a CNV measure. We used the technical zero baseline approach for the CNV, which measures the absolute state rather than the amount of neural change introduced by the event, and has been used in previous CNV work (Albrecht et al. 2013; Banaschewski et al. 2003). We chose this short interval as it captures the late CNV component unconfounded by sensory activity and characterized by a typical CNV topography in the fast-incentive condition with its 1-s cue–target interval (Fig. 2c, d; no typical CNV topography emerged in the slower baseline condition). We used the same corresponding time-window in the baseline condition to examine within-subject change in preparatory activity across conditions.

### Statistical Analyses

All initial group analyses included IQ as a covariate; we subsequently re-ran the analyses without IQ as a covariate. RTV data were skewed and transformed using the optimized minimal skew (lnskew0) command in Stata (Stata Corporation, College Station, TX). As these were sibling data, the data were analysed using random intercept models and logistic regression in Stata. The random intercept model is a multilevel regression model that can be used as an alternative to ANCOVA to control for genetic relatedness in a repeated-measures design, using a “robust cluster” command to estimate standard errors (Tye et al. 2012; Wood et al. 2009), as the robust cluster command is not available for ANOVA.

We first computed the group differences in both the baseline and the fast-incentive condition, followed by a post-hoc group analysis of the difference score between conditions. As the RTV difference scores could not be successfully transformed, non-parametric Kruskal–Wallis tests were used on the singletons, by removing the siblings from the ADHD (*n* = 8) and control (*n* = 81) groups. Singletons were randomly drawn from complete sibling pairs using the “sample” command in Stata. The robust cluster command was not available in the correlational analysis, so for these analyses only the singletons (n = 194) were included. As the CNV was maximal at Cz, we computed the correlations with the CNV at Cz only, to reduce the number of statistical comparisons.

### Additional Analyses

To rule out that the P3 findings are not a result of spatial overlap with the anterior positive CNV signals, we applied a CSD transformation to ‘sharpen’ the scalp topography and reduce the spatial overlap of these two functionally distinct signals (Kayser and Tenke 2006; Kelly and O’Connell 2013). To directly examine the effect of CNV on P3 amplitudes, we conducted additional analyses of P3 with CNV at Pz included as a covariate. This analysis enabled us to take into account and statistically control for the influence group effects that are already present during the baseline intervals, comparable to adding an analysis using a technical zero baseline approach without baseline subtraction or correction for the P3 (Koenig and Gianotti 2009; Maess et al. 2016). To rule out that our P3 interpretations were not due to group differences in a later post-stimulus interval or in the corresponding response-aligned ERP (MRT = 575 ms in the baseline condition and 442 ms in the fast-incentive condition), we conducted separate analyses with a later interval of the P3 (between 450 and 600 ms at Pz, Figure S1), along with a response-aligned ERP measured as the area amplitude measures (μV*ms) at Pz at the −250 to −100 ms interval before the response onset (Kelly and O’Connell 2013). These results are presented in the online supplementary materials.

## Results

### RTV

**Fig. 1:**
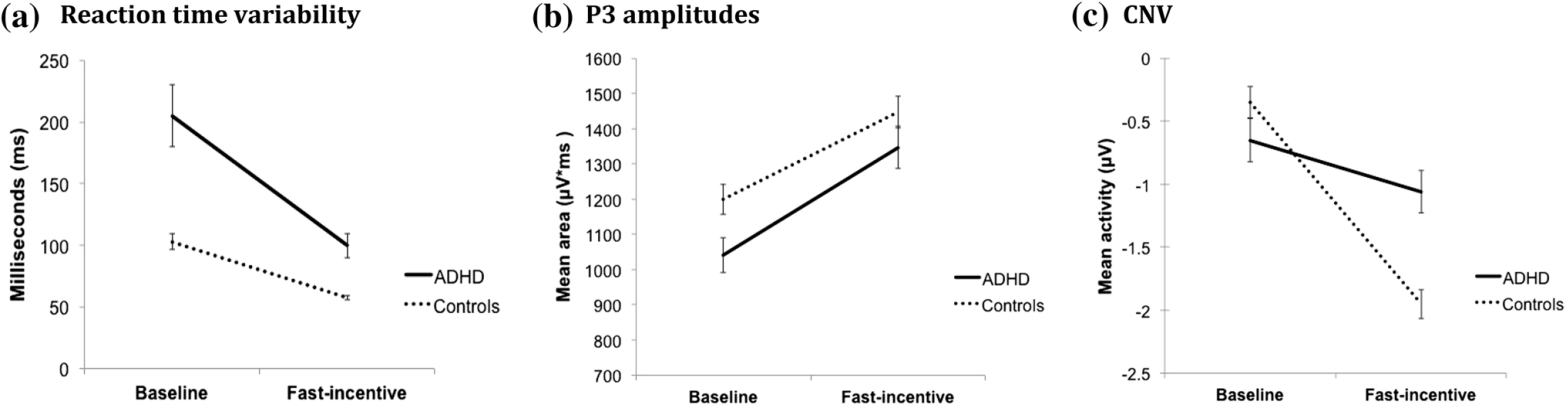
ADHD-control comparisons on **a** reaction time variability, **b** P3 amplitudes and **c** prestimulus CNV acitivity across baseline and fast-conditions of the Fast Task, including SEM error bars

A random intercept model indicated significant main effects of group (z = 4.77, p < 0.01) and condition (z = −10.29, p < 0.01) for RTV, and a significant group-by-condition interaction (z = −2.44, p = 0.02) (Fig. 1a). Post-hoc regression analyses indicated increased RTV in individuals with ADHD compared to controls in the baseline (z = 5.30, p < 0.01) and fast-incentive (z = 3.44, p < 0.01) conditions, with the change between the conditions significantly greater in the ADHD than control group (χ^2^(1) = 18.29, p < 0.01). We obtained comparable results using the length-matched segment of the baseline condition (Andreou et al. 2007) where individuals with ADHD displayed a significantly increased RTV in the baseline condition (z = 3.92, p < 0.01) and a significantly greater improvement in the fast-incentive condition (χ^2^(1) = 12.68, p < 0.01), compared to the controls.

### P3 Amplitudes

**Fig. 2:**
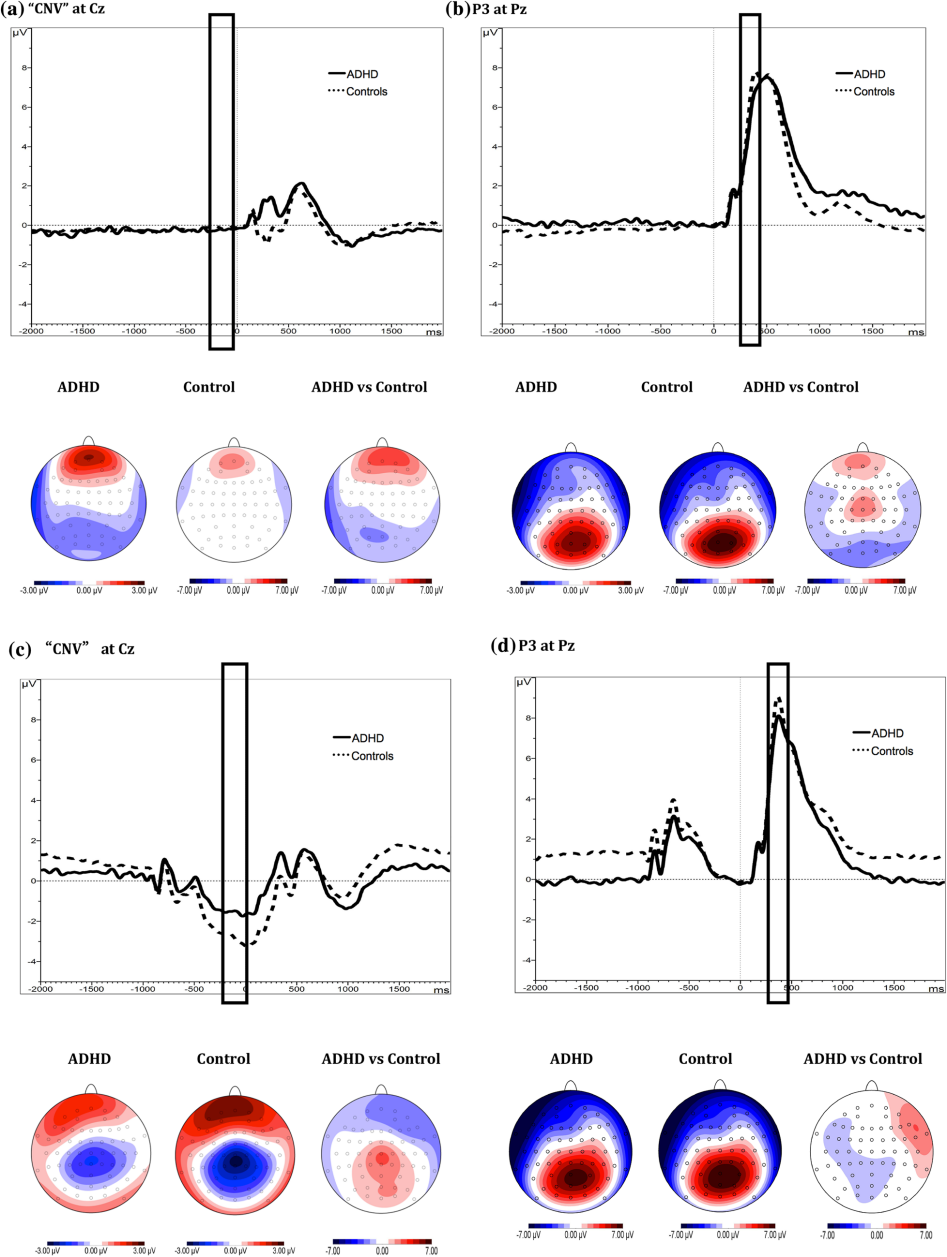
Topographic maps, t-maps and waveforms for the CNV and P3. *Black rectangles* mark the time windows of CNV amplitudes at Cz (*left column* **a, c**, with no baseline correction), and of P3 amplitudes at Pz (*right column*, **b, d**, with −200 to 0 ms prestimulus baseline correction). *Solid lines* represent the ADHD group and *dotted lines* represent the control group in the baseline (*top* **a, b**) and fast-incentive (*bottom* **c, d**) condition of the Fast Task

A random intercept model showed main effects of group (z = −2.16, p = 0.03) and condition (z = 45.25, p < 0.01) for P3 amplitudes, but the group-by-condition interaction did not reach significance (z = 1.22, p = 0.22) (Figs. 1b, 2b). Post-hoc tests were not performed, but the raw mean values for P3 amplitudes across the two conditions are presented (Table S3). A second P3 peak was observed in the baseline condition (Fig. 2b), and we therefore carried out an additional analysis on this later time window (450–600 ms). No significant effects of group or group by condition were observed for this measure (Figure S1). We ran a separate analysis on the response-aligned ERP to determine if there are group effects reflecting neural decision threshold. Again, no significant effects of group or group by condition were observed (Figure S2).

### CNV

A random intercept model showed an overall trend effect of group at Cz (z = 1.88, p = 0.06) but not at Pz (z = 0.04, p = 0.97). The main effect of condition was significant at both Cz and Pz (z = −15.65, p < 0.01; z = −9.07, p < 0.01, respectively). The group by condition interaction was also significant at Cz (z = 4.44, p < 0.01) and Pz (z = 3.95, p < 0.01). Post-hoc comparisons revealed no group differences on the CNV in the baseline condition at Cz (Figs. 1c, 2a) or Pz (z = −1.37, p = 0.17; z = −1.53, p = 0.13, respectively), but in the fast-incentive condition the control group exhibited greater amplitude of the CNV compared to the ADHD group at Cz (Fig. 2c, z = 3.53, p < 0.01) and Pz (z = 1.91, p = 0.06). The change in CNV amplitudes across conditions was significantly greater in the control than the ADHD group at both Cz and Pz (z = −4.00, p < 0.01; z = −2.59, p = 0.01, respectively).

### P3 CSD Amplitudes

A random intercept model showed main effects of group (z = −2.32, p = 0.02) and condition (z = 13.39, p < 0.01) for P3 amplitudes, with the group-by-condition interaction just reaching significance (z = 1.93, p = 0.05) (Figure S3). Post-hoc comparison revealed significant group differences in the baseline (z = −2.87, p < 0.01) but not in the fast-incentive (z = −1.14, p = 0.26) condition.

### CNV Effects on P3 Amplitudes

To confirm that the P3 group by condition effect observed following CSD transformation was due to the CNV, we re-ran the analysis on P3 including CNV amplitude at Pz as a covariate, both with CSD transformation. This covariate approach allows us to take into account the group effects during the baseline intervals, similar to using a technical zero baseline approach for the P3 in which a significant group by condition interaction for P3 was observed (see Figure S4). With CNV amplitudes included in the same model, a group by condition interaction now emerged for the P3 (z = 2.01, p < 0.05), with the main effects of group and condition remaining significant (z = −2.56, p = 0.01; z = 14.03, p < 0.01, respectively). Post-hoc analyses indicated that the ADHD group showed significantly reduced P3 amplitudes compared to the control group in the baseline condition (z = −2.95, p < 0.01) but not in the fast-incentive condition (z = −1.69, p = 0.09).

All analyses were re-run without IQ as a covariate, and the pattern of results remained the same with two exceptions. First, the main effect of group for P3 amplitudes diminished (z = −1.63, p = 0.10). Further examination of the effects of IQ on P3 amplitudes was, therefore, carried out on the ADHD and control groups separately. A negative association between IQ and P3 amplitudes was observed in the control group in the baseline condition (r = −0.21, p = 0.04), but not in the fast-incentive condition (r = 0.03, p = 0.79). In the ADHD group, IQ and P3 amplitudes were not significantly correlated in the baseline condition (r = −0.10, p = 0.38) but were associated at trend level in the fast-incentive condition (r = 0.20, p = 0.07). Second, the magnitude of group difference in the CNV at Pz in the fast-incentive condition changed from a trend (p = 0.06) to statistical significance (z = 3.41, p < 0.01) when IQ was not controlled for. The latter finding suggests that IQ differences between ADHD and control group may account for the group differences in the CNV at Pz.

### Relationship Between RTV, P3 Amplitude and CNV

As age correlated significantly with P3 in both groups (Table S2), and RTV varied as a function of gender in the ADHD group, they were included as covariates in the within-group correlational analyses, in addition to IQ. In the ADHD group, a significant association was observed between RTV and P3 amplitudes and between CNV and P3 amplitudes in both the baseline and fast-incentive conditions, while a significant association between RTV and CNV was only observed in the fast-incentive condition (Table 1). In the control group, a significant association was observed between RTV and P3 amplitudes only in the fast-incentive condition. While among controls no association was observed between CNV and P3 amplitudes in either condition, a significant association between RTV and CNV was observed in both conditions. Fisher’s z test of significance between two correlation coefficients indicated that, in the baseline condition, the correlation between P3 and RTV tended to be higher in the ADHD than control group (z = −1.58, p = 0.06). The group differences were not significant for the P3-RTV correlation in the fast-incentive condition, for the CNV–RTV or for the CNV-P3 correlations in either condition (p = 0.10–0.44).

**Table 1:** Pearson correlations (two-tailed) between P3 amplitude, contingent negative variation (CNV) at Cz and reaction time variability (RTV), in the baseline and in the fast-incentive conditions, controlling for effects of age, gender and IQ

	Baseline		Fast-incentive	
	ADHD	Control	ADHD	Control
RTV and P3	−0.32**	−0.09	−0.23*	−0.21*
RTV and CNV	0.18	0.36**	0.36**	0.29**
P3 and CNV	−0.22*	−0.17	−0.23*	−0.09

*p = 0.05

**p < 0.01

Correlations of the difference scores across conditions indicate that a reduction in RTV from the baseline to fast-incentive condition was significantly associated with an increase in P3 amplitudes in the ADHD group, whereas this pattern was not observed in the control group, with the difference in the correlation coefficients reaching significance between groups (p < 0.01; Table 2). Among controls an increase in P3 amplitudes across the two conditions was associated with an increase in CNV. This correlation was weaker in the ADHD group with a trend-level group difference (p = 0.08; Table 2). The magnitude of change in CNV was unrelated to the degree of change in RTV in either ADHD or control group, and the correlation coefficients were not different between two groups (Table 2). We then re-ran the correlations without controlling for the effects of IQ, and the overall pattern of results remained similar with one exception: the correlation between RTV and CNV in the ADHD group in the baseline condition became significant (r = 0.30, p < 0.01) (Table S4).

**Table 2:** Pearson correlations (two-tailed) between the change scores (baseline vs fast-incentive conditions) of P3 amplitude, contingent negative variation (CNV) and reaction time variability (RTV), controlling for effects of age, gender and IQ. Fisher’s z test of significance between two correlation coefficients

	ADHD	Controls	z	p
P3 and CNV	0.01	0.22*	−1.40	0.08
CNV and RTV	−0.09	−0.04	−0.33	0.37
P3 and RTV	0.28*	−0.12	2.67	<0.01

*p = 0.05

## Discussion

We show, first, that ADHD is associated with attenuated P3 amplitudes during performance on an RT task, indicating difficulties with attentional resource allocation. Second, the main neurophysiological measure of attention (P3) is linked to the cognitive performance measure of attentional fluctuation (RTV) in individuals with ADHD, with both measures showing malleability: attenuated P3 amplitudes were significantly associated with high RTV, and the increase in P3 amplitudes from a baseline to a fast-paced, rewarded condition was significantly associated with the decrease in RTV observed between conditions. Yet, third, the individuals with ADHD did not show the same increase in CNV from baseline to fast-incentive condition as observed in controls, indicating that they were unable to adequately adjust the preparatory state in a changed context.

Our findings on RTV replicate those reported in the previous study on a partially overlapping group of children performing the identical Fast task 6 years earlier (Andreou et al. 2007), confirming that while attentional fluctuation in ADHD is malleable—showing potential for improvement—it is also a developmentally stable and persistent trait in individuals with current diagnoses of ADHD. As far as we are aware, only a few studies have, separately, investigated the effects of stimuli presentation speed (Wiersema et al. 2006a, b) and incentives (Groom et al. 2010) on the P3 component in ADHD. This study is therefore the first to combine the two task manipulations to investigate its effect on both neurophysiological and behavioural levels, and to examine the relationship between these traits, which are both associated with ADHD. We extend previous findings of associations between P3 amplitudes and performance variability in population-based samples (Ramchurn et al. 2014; Saville et al. 2011) to a group of adolescents and adults with current ADHD. The malleability of the neurophysiological marker of attention induced by a faster paced and rewarded condition supports future investigation of non-pharmacological interventions that target moderating P3 amplitudes and emphasises the advantages of incorporating fast-paced activities and incentives in the learning environments for individuals with ADHD.

Considering our findings on P3 amplitudes in more detail, the group by condition interaction effect only emerged as significant when CSD transformation was applied, and when CNV activity was included in the model. Both these results converge to suggest that anterior signals generated by pre-stimulus preparatory activity lead to ‘contamination’ of posterior P3 signal. Sharpening the topography of these signals revealed a group by condition interaction effect. In a further examination of the relationship between CNV and P3 we showed that the ADHD-specific improvement in attention allocation is partially obscured when not considering preceding differences in preparatory activity. This additional analysis on the pre-target activity (CNV) suggests that the lack of group by condition interaction effect in P3 may be partially accounted for by subtracting the more prominent increase in CNV in controls than in the ADHD group during the fast-incentive condition as the baseline. Thus, the preparatory brain processes in individuals with ADHD were not comparable to controls in the fast-rewarded condition. As RTV improves under the fast-incentive condition, we observed a significantly greater magnitude of change in preparatory CNV activity from the baseline to the fast-incentive condition among controls compared to individuals with ADHD. The overall pattern of findings suggests that the inability to adjust the preparatory state in a changed context may explain why RTV does not fully normalise in ADHD. These findings also highlight the importance of considering the effects of pre-stimulus state measures, such as the CNV, when interpreting the findings on post-stimulus ERPs, particularly in individuals with ADHD. Studies that subtract the pre-stimulus baseline ERP activity from the post-stimulus ERPs without testing for systematic effects during this pre-stimulus period may risk overlooking crucial neurophysiological processes that underlie behavioural states and their influence on subsequent components, which could lead to misinterpretations.

Little is known about how attentional processes on a neural level relates to attentional fluctuation on a cognitive performance level, even amongst the general population. The results from our control group indicate that, under a slow and unrewarded baseline condition, RTV relates more strongly with preparation (CNV) than with allocation of attention (P3 amplitudes). By comparing the associations between these ERP markers with RTV across conditions, we further demonstrate that for individuals with and without ADHD, both attentional resource allocation (P3 amplitudes) and response preparation (CNV) are necessary for optimal cognitive performance in the fast-incentive condition. As the main effects of condition were significant for both P3 and CNV amplitudes, it suggests that regardless of ADHD status, attentional allocation and response preparation are both contributing factors for optimal cognitive performance in the fast-incentive condition. This finding is consistent with a recent study that the prospect of gaining monetary reward increased CNV amplitudes and reduced reaction time in healthy adults (van den Berg et al. 2014). The role of attentional preparation in reaction time performance is further supported by a recent study that found methylphenidate to concurrently increase CNV amplitudes and reduce reaction time variability (Linssen et al. 2011). As methylphenidate acts on the catecholaminergic pathways, these findings indicate that the CNV is a catecholaminergic system marker that plays a critical role in information processing in the general population (van den Berg et al. 2014), in individuals with ADHD (Albrecht et al. 2013) as well as other disorders (Dhar et al. 2010). Our findings also corroborate with a recent report that showed the role of pre-target attentional state in augmenting neural decision-making process to influence behaviour (Kelly and O’Connell 2013). We also included a supplementary response-aligned P3 measure to disentangle different aspects of post-stimulus information processing in relation to ADHD. The results suggest that in this particular task, individuals with ADHD are different from controls only at a level of attentional engagement rather than at later response-related decision-making levels.

Our findings remained largely consistent whether or not IQ was included as a covariate, except for two patterns of findings. Firstly, the association between CNV and RTV in the ADHD group only emerged when IQ was not included as a covariate and only in the fast-incentive condition, suggesting that individual differences in IQ may account for the relationship between CNV and RTV in ADHD, under specific task circumstances. Secondly, the ADHD-control group differences on P3 amplitudes were only significant when IQ was included as a covariate. One explanation for this could be that IQ plays a differential role on P3 amplitudes among controls and individuals with ADHD: while we observed a significant negative association between IQ and P3 amplitudes, this association was absent among participants with ADHD. Although prior studies using the go/no-go task have reported positive associations of P3 amplitude with IQ (Dichter et al. 2006) and with academic achievement (Hillman et al. 2012) in the general population (Dichter et al. 2006), this is the first study to report the relationship between P3 amplitude and IQ using a simple choice reaction time task. Our findings indicate that the extent of attentional resources required to process stimuli in a slow and unrewarded task decreases as a function of IQ in typically developing individuals. Future studies should further examine whether the negative association between P3 amplitude and IQ in controls is driven by the use of alternative neural mechanisms.

A limitation of this study was that, while the two task conditions were matched on the number of trials, they differed in task length and we were unable to perform additional ERP analyses on length-matched segments due to insufficient number of trials. As such, while our findings illustrate how attentional performance can be improved in ADHD, future studies are needed to investigate how such improvements can be maintained longer term. At the performance level (RTV), we obtained identical findings whether or not length-matched segments were used. The design of the Fast Task paradigm is not ideal for measuring a conventional CNV, as the interval between the cue is very long in the baseline condition and relatively short for the fast-incentive condition. In spite of the short time interval in the latter condition, we observed a typical CNV distribution in the fast condition, and a prominent group difference in this preparatory activity, suggesting that CNV is a sensitive marker of ADHD also at short intervals. Future studies could replicate these findings regarding anticipatory activity using other ERP tasks. Due to the long inter-stimulus interval of our task design, we were unable to obtain an accurate latency measure. However, as previous studies suggest, both amplitude and latency of the stimulus-aligned P3 are dependent on RT distribution (O’Connell et al. 2012). Future single-trial analyses are therefore needed to confirm that our P3 findings reflect true diminution of signal at the single-trial level, rather than simply reflecting increased RTV. Future single-trial analyses in this area will also be invaluable to clarify the chronology of events and to test any upstream influence of P3 on CNV activity.

Overall, our findings provide novel insights into the neurophysiological basis of the attentional fluctuation observed as high RTV in adolescents and young adults with ADHD. Our results demonstrate the potential of inducing an optimal task condition to improve attention on both neurophysiological and behavioural levels. While individuals with ADHD reduced attentional fluctuation by increasing their attention allocation, they were unable to adjust their preparatory response appropriately in a changed context. Consistent with our previous genetic model fitting finding that RTV baseline performance and its improvement across conditions measure the same aetiological process (Kuntsi et al. 2012), findings from this study show that the same neurophysiological process underlies RTV baseline performance and its improvement in ADHD. Although this and previous studies suggest that attenuated P3 amplitudes and increased RTV are developmentally stable markers of ADHD in those with persisting ADHD diagnosis (Cheung et al. 2016; Szuromi et al. 2011), both show malleability and are therefore targets for non-pharmacological interventions.

## Electronic supplementary material

Below is the link to the electronic supplementary material.

Supplementary material 1 (DOCX 3961 KB)
