# Supplementary material for: Neurophysiological Correlates of Attentional Fluctuation in Attention-Deficit/Hyperactivity Disorder
Source: Brain Topogr. 2017 Mar 14;30(3):320–32. doi: 10.1007/s10548-017-0554-2 (PMC5408051; doi:10.1007/s10548-017-0554-2)

**Table S1.** Group comparisons on age, gender and IQ.

|  | **ADHD** | **Control** | ***t/x*** | ***p*** |
| --- | --- | --- | --- | --- |
| Age range  Mean age (sd) | 12-22  18.83 (2.98) | 12-26  17.75 (2.14) | -  -1.87 | -  0.06 |
| Gender (male %) | 77% | 84% | 1.88 | 0.17 |
| IQ | 96.27 (15.06) | 109.42 (12.57) | 7.69 | <0.01 |

**Table S2.** Pearson correlations (two-tailed) of age with RTV, P3 and CNV in the baseline and fast-incentive conditions.

| Correlation with age | ADHD | Control | ADHD | Control |
| --- | --- | --- | --- | --- |
| RTV | -0.10 | -0.29** | -0.06 | -0.17* |
| P3 | -0.45** | -0.26** | -0.37** | -0.32** |
| CNV | -0.17 | 0.02 | -0.16 | 0.05 |

**** p<0.01, * p=0.05**

**Table S3.** Means and standard deviations for RTV, P3 and CNV in the baseline and fast-incentive conditions.

|  | **Baseline** | | **Fast-incentive** | |
| --- | --- | --- | --- | --- |
|  | ADHD | Control | ADHD | Control |
| RTV | 205.08 (240.25) | 102.95 (82.75) | 99.66 (95.48) | 57.22 (20.53) |
| P3 | 1041.119 (473.93) | 1199.09 (553.66) | 1346.62 (568.74) | 1447.88 (592.57) |
| CNV | -0.65 (1.63) | -0.35 (1.68) | -1.06 (1.59) | -1.95 (1.52) |

**Table S4.** Pearson correlations (two-tailed) between P3, CNV and RTV in the baseline and in the fast-incentive conditions, controlling for effects of age, gender but not controlling for IQ.

|  | **Baseline** | | **Fast-incentive** | |
| --- | --- | --- | --- | --- |
|  | ADHD | Control | ADHD | Control |
| RTV and P3 | -0.32** | -0.04 | -0.36** | -0.22* |
| RTV and CNV | 0.30** | 0.38** | 0.38** | 0.36** |
| P3 and CNV | -0.22* | -0.14 | -0.25* | -0.10 |

**** p<0.01, * p=0.05**

**Figure S1.** Topographical and t-maps of the late-P3 (450-600ms) in the ADHD and control groups in the baseline and fast-incentive conditions. A significant main effect emerged for condition (*z*=-26.72, *p*<0.01), but not for group (*z*=-0.30, *p*=0.76). Group by condition interaction was not significant (*z*=0.57, *p*=0.57).


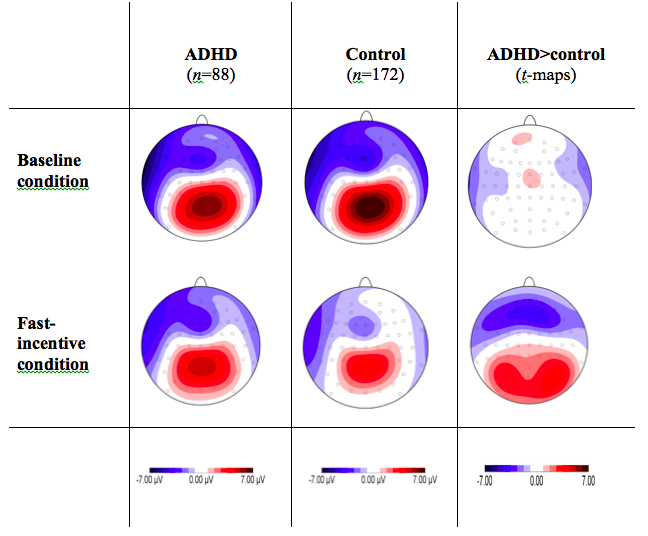


**Figure S2.** ERP waveforms and topographical maps of the response-aligned average at Pz (-250-100ms before response onset corresponding best to the stimulus-aligned P3 time window) in the ADHD (solid line) and control (dotted line) groups in the baseline and fast-incentive conditions. The topographic maps indicate the same posterior component as the stimulus-aligned P3. A significant main effect emerged for condition (z= -13.14, p<0.01), but not for group (z=0.92, p=0.36). Group by condition interaction was not significant (z=1.33, p=0.19).

**
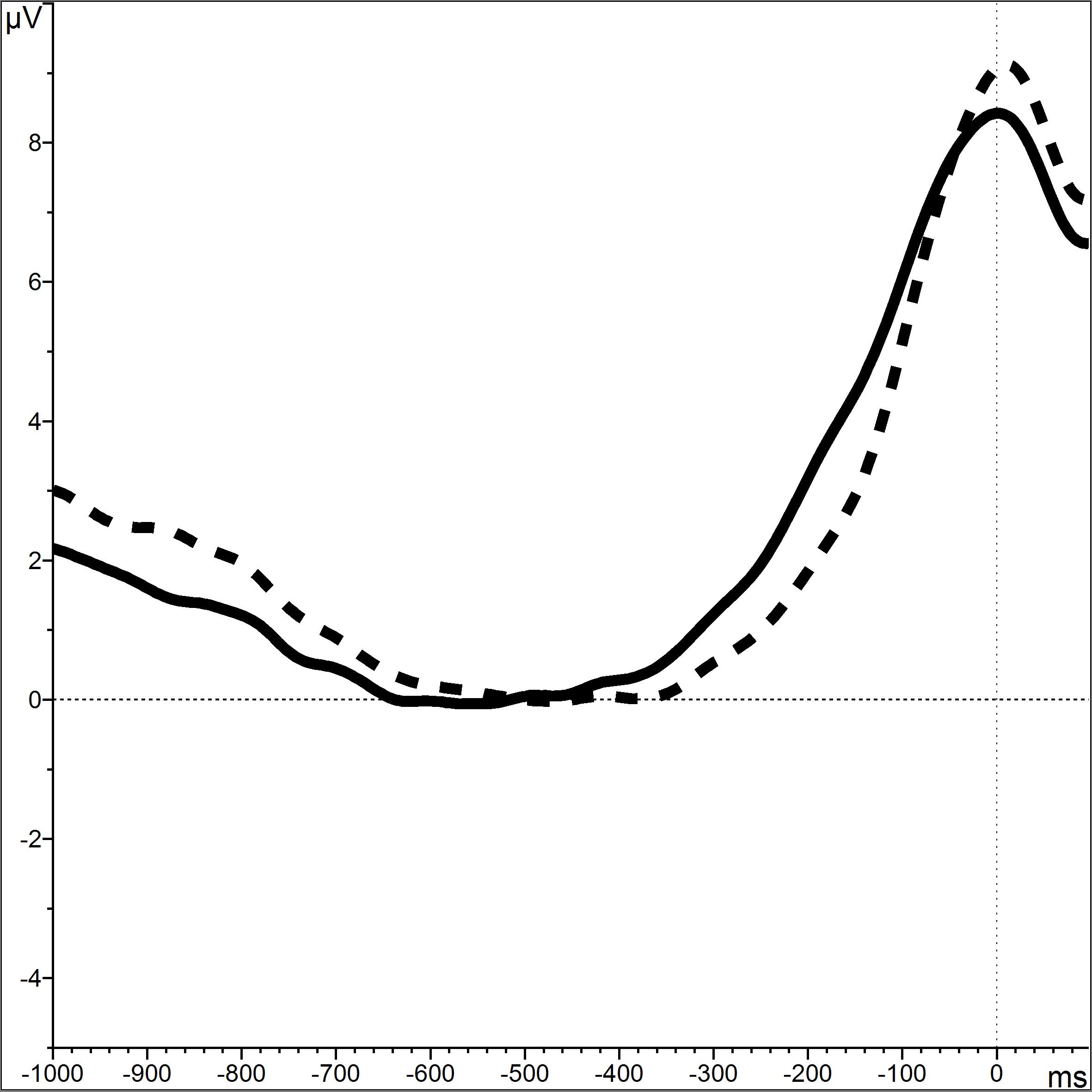

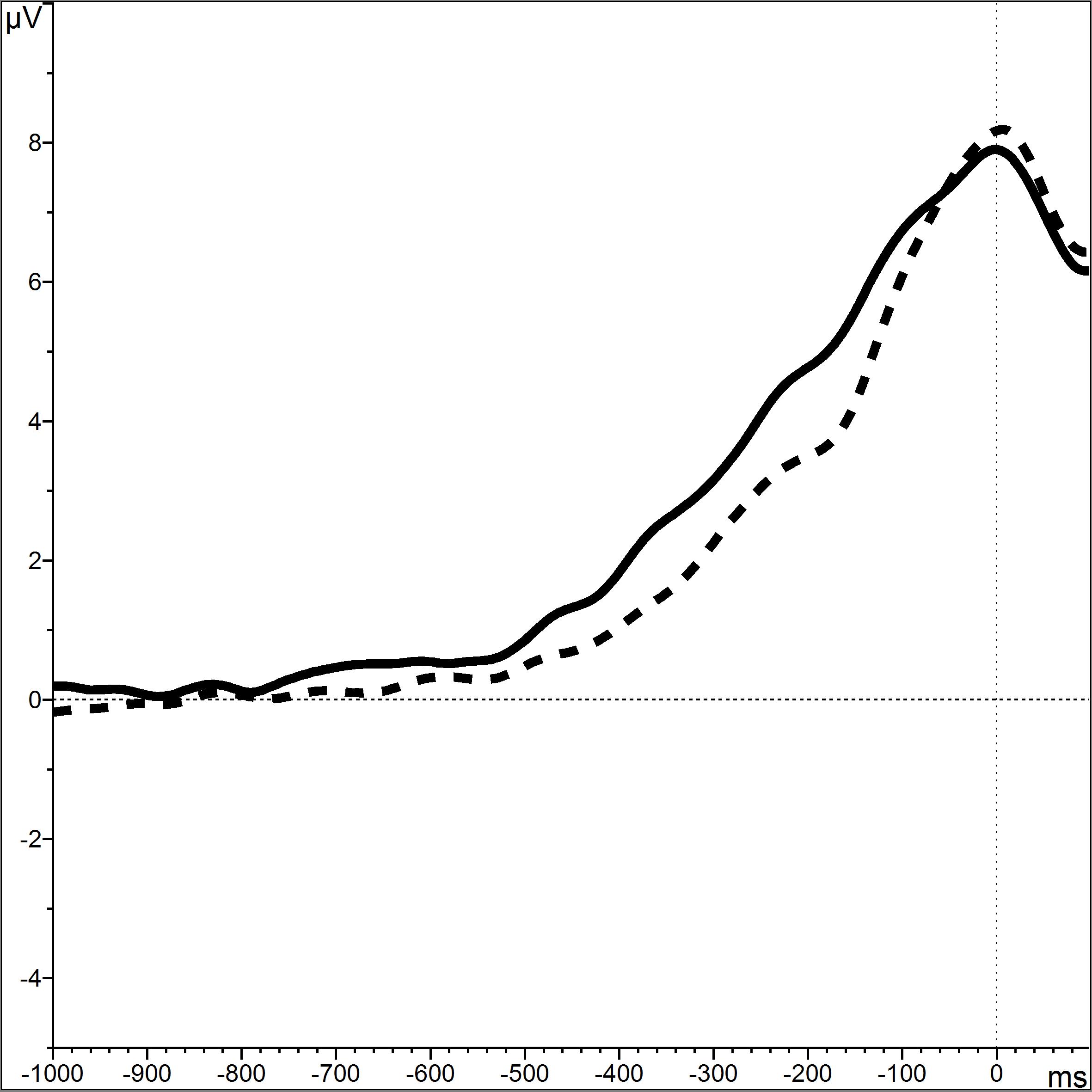
**

Fast-incentive

Baseline

**ADHD Control**

**ADHD Control**


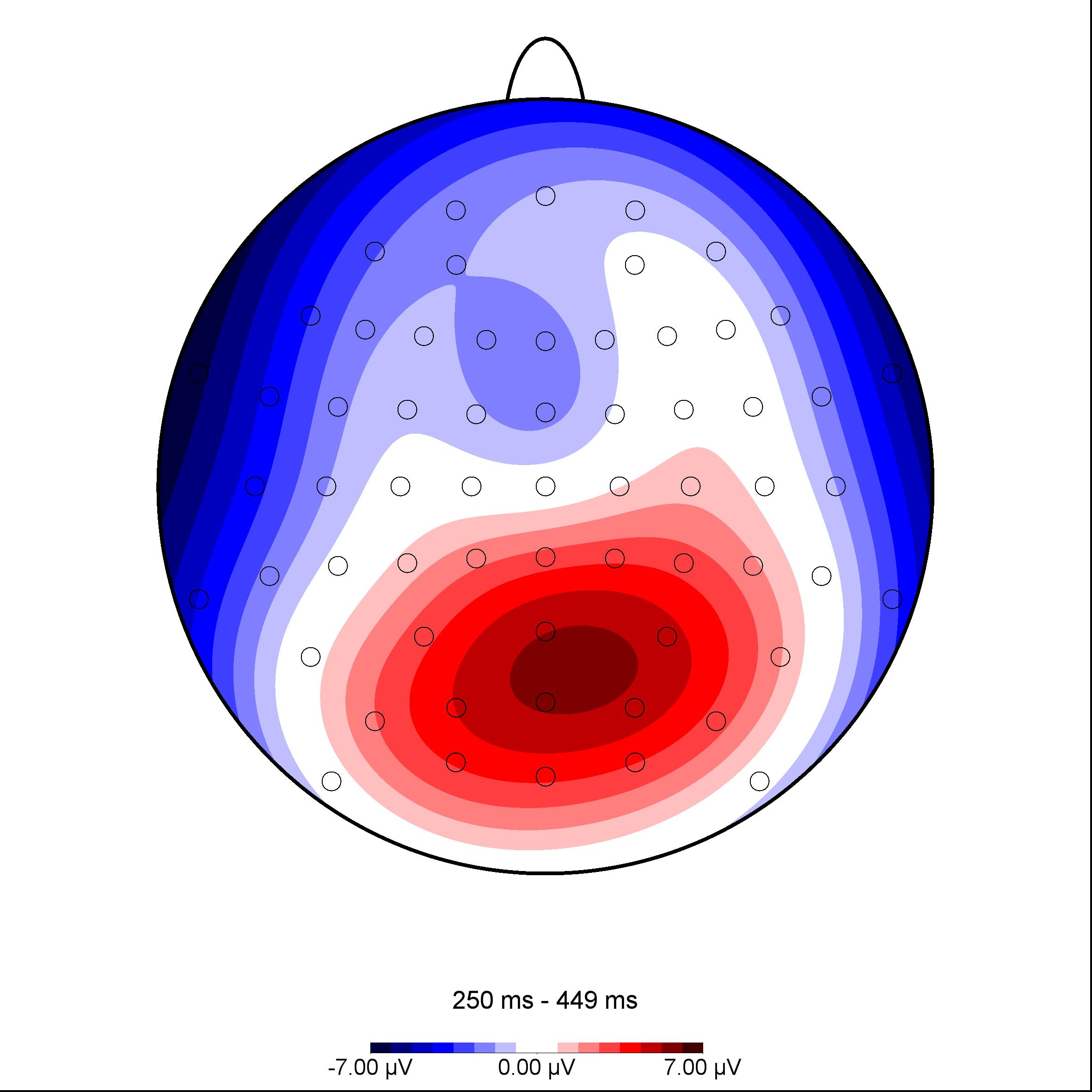

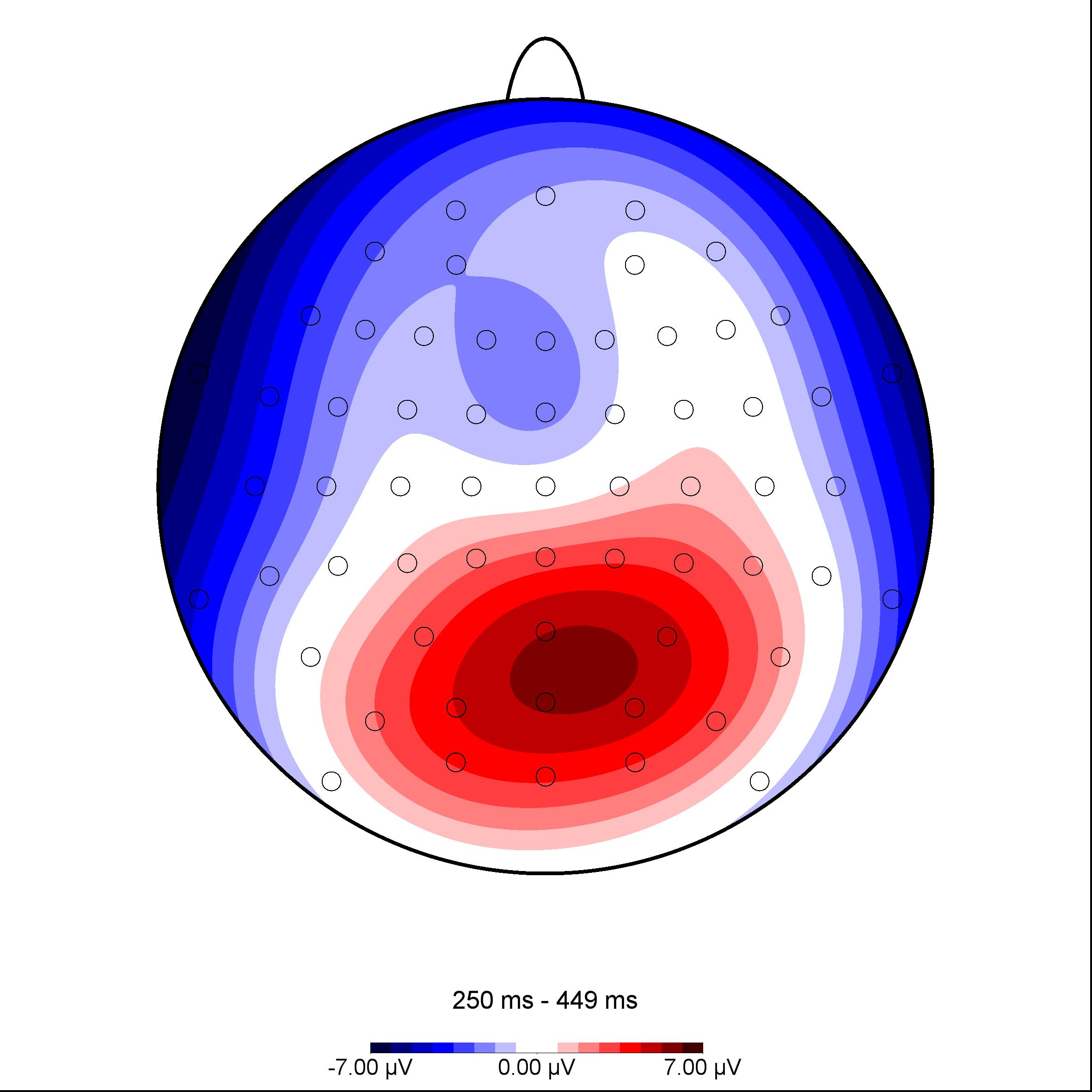

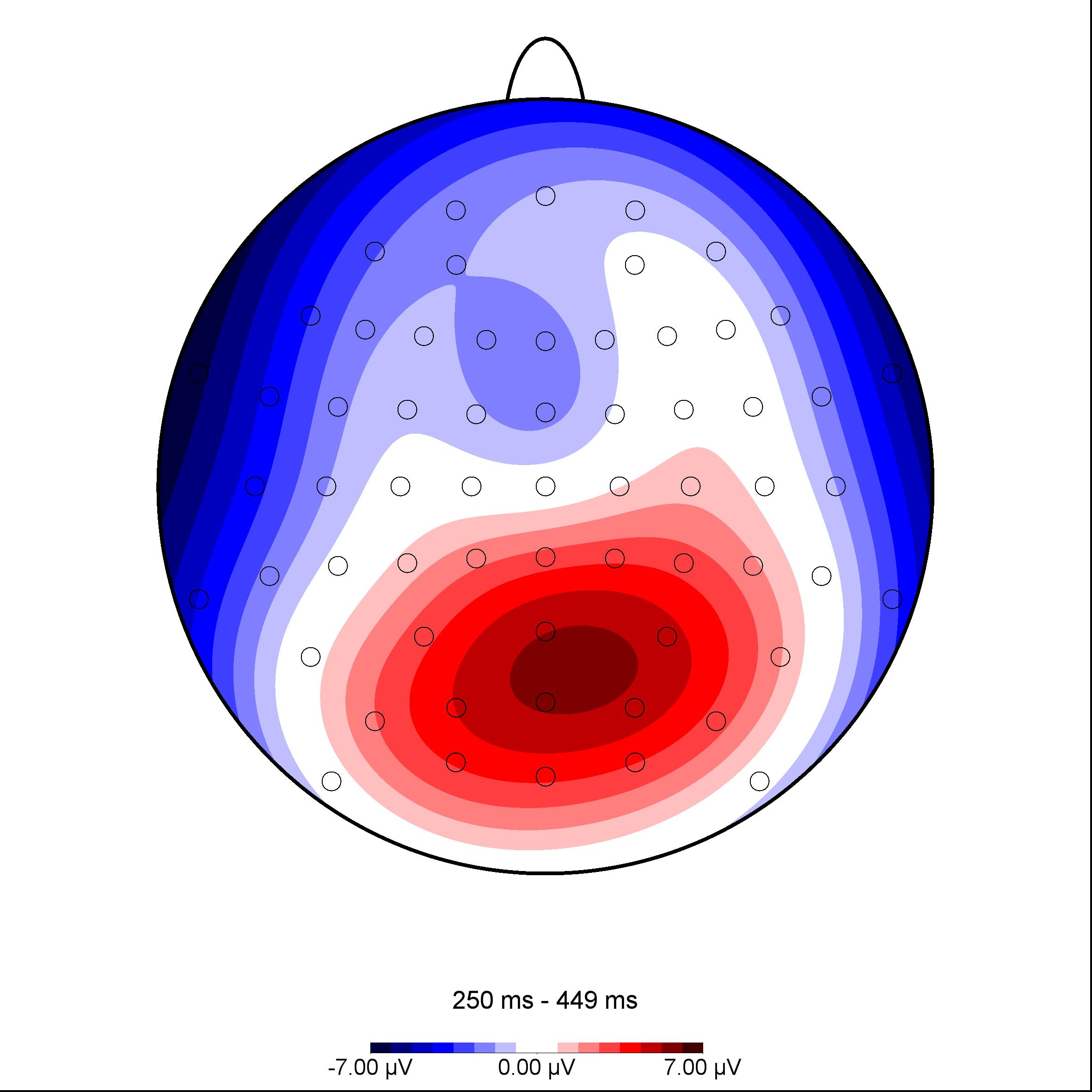

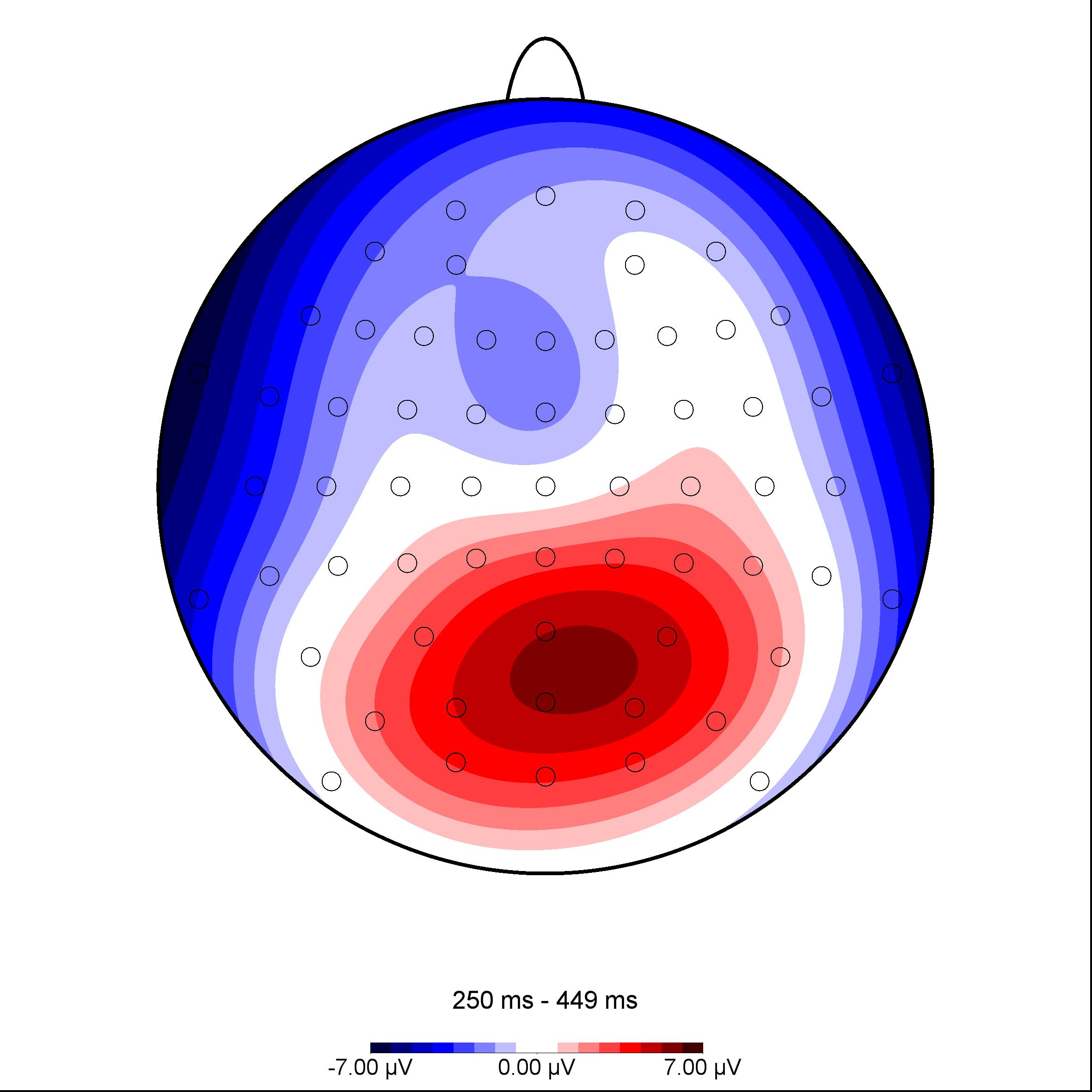
**Figure S3.** CSD waveform and maps of the average P3 at Pz after CSD transformation of the ERPs in the ADHD (solid line) and control (dotted line) groups in the baseline and fast-incentive conditions.

| 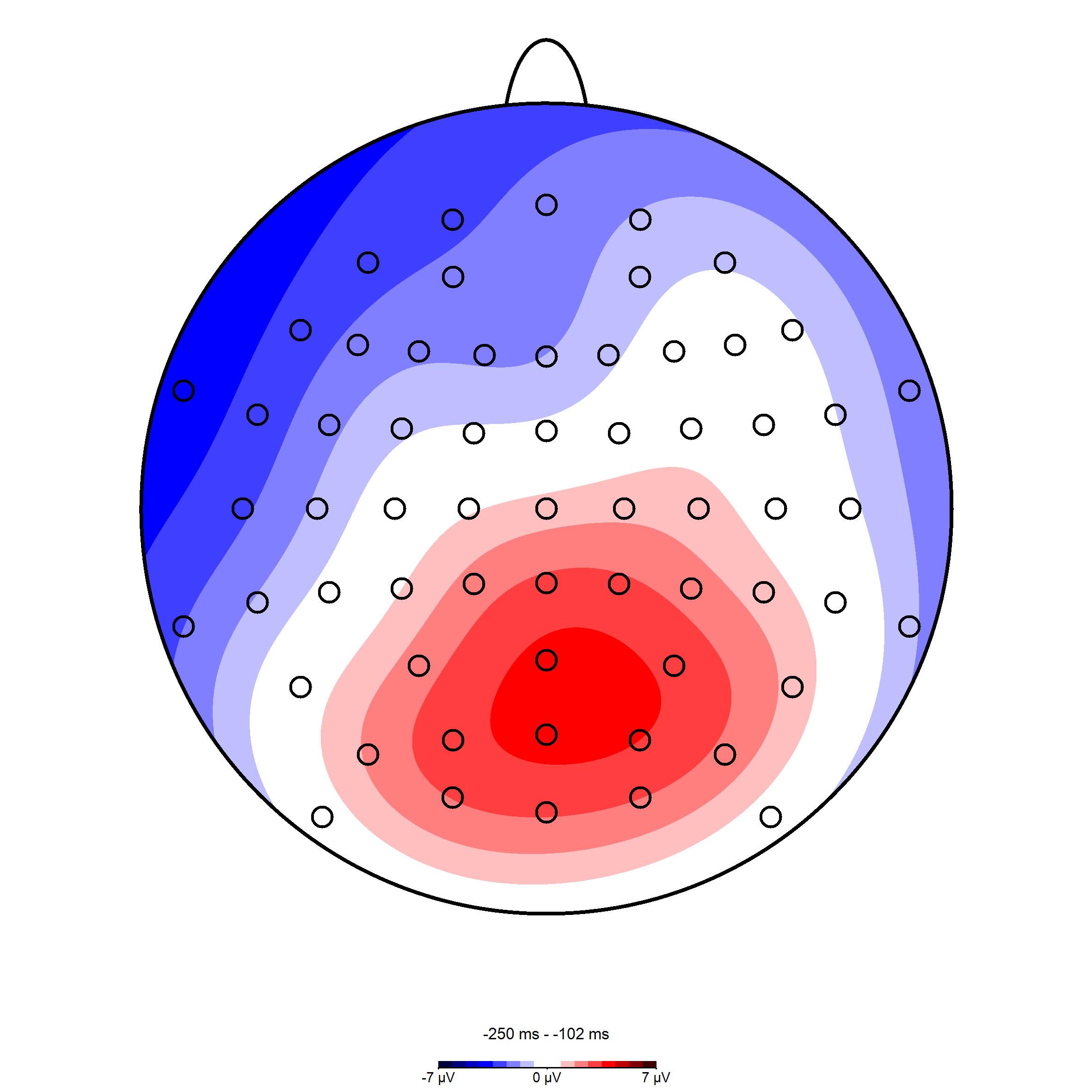 | 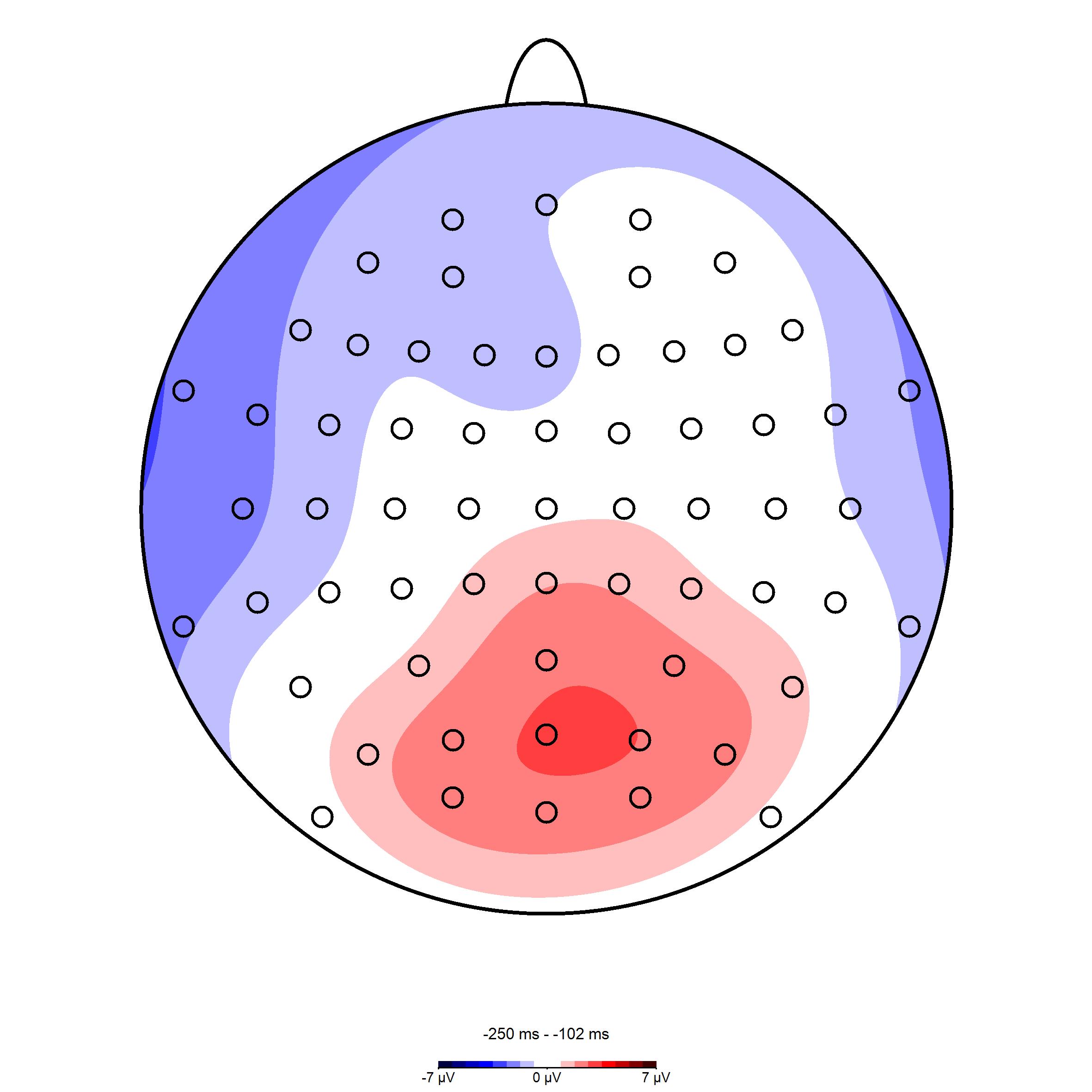 |  |
| --- | --- | --- |

| 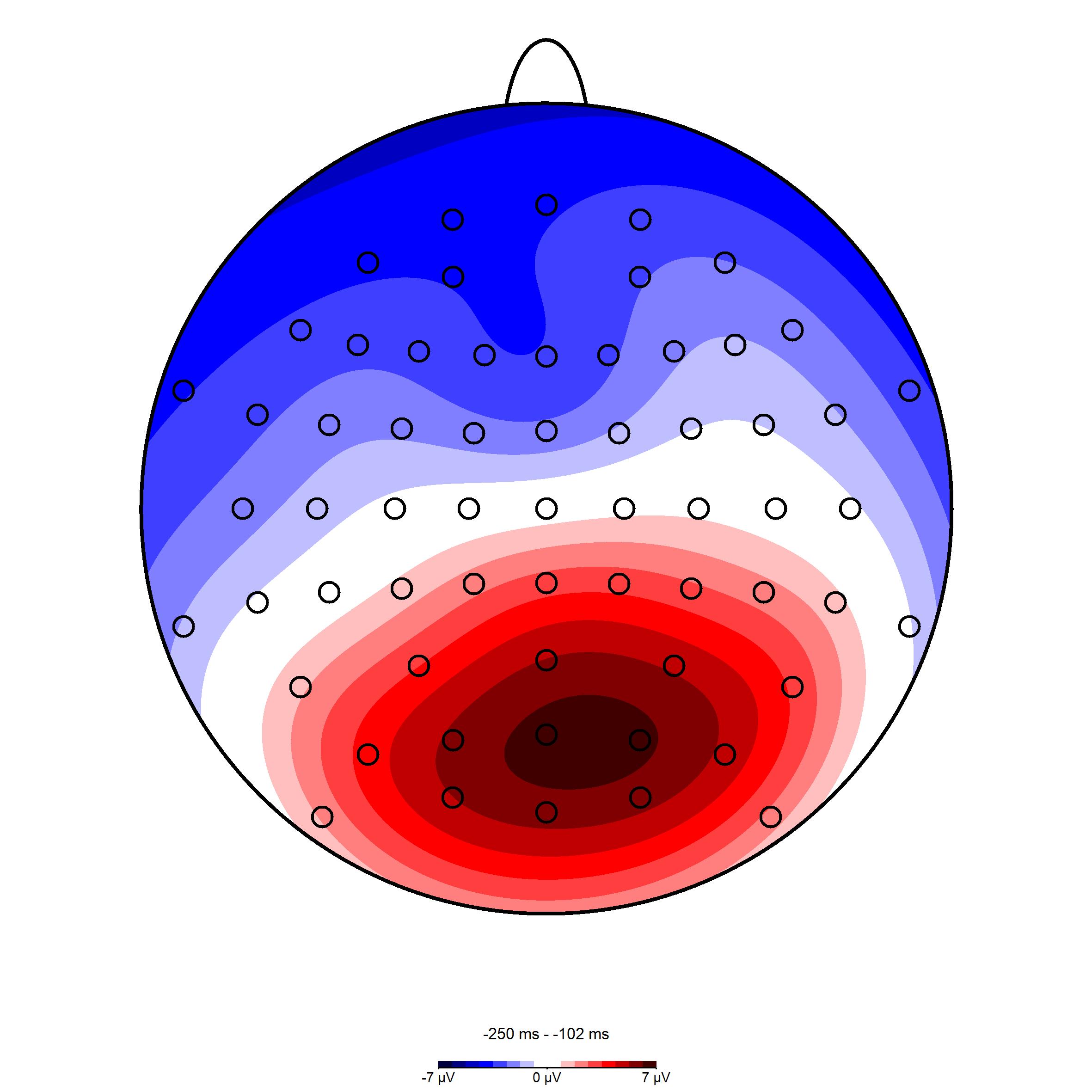 |  | 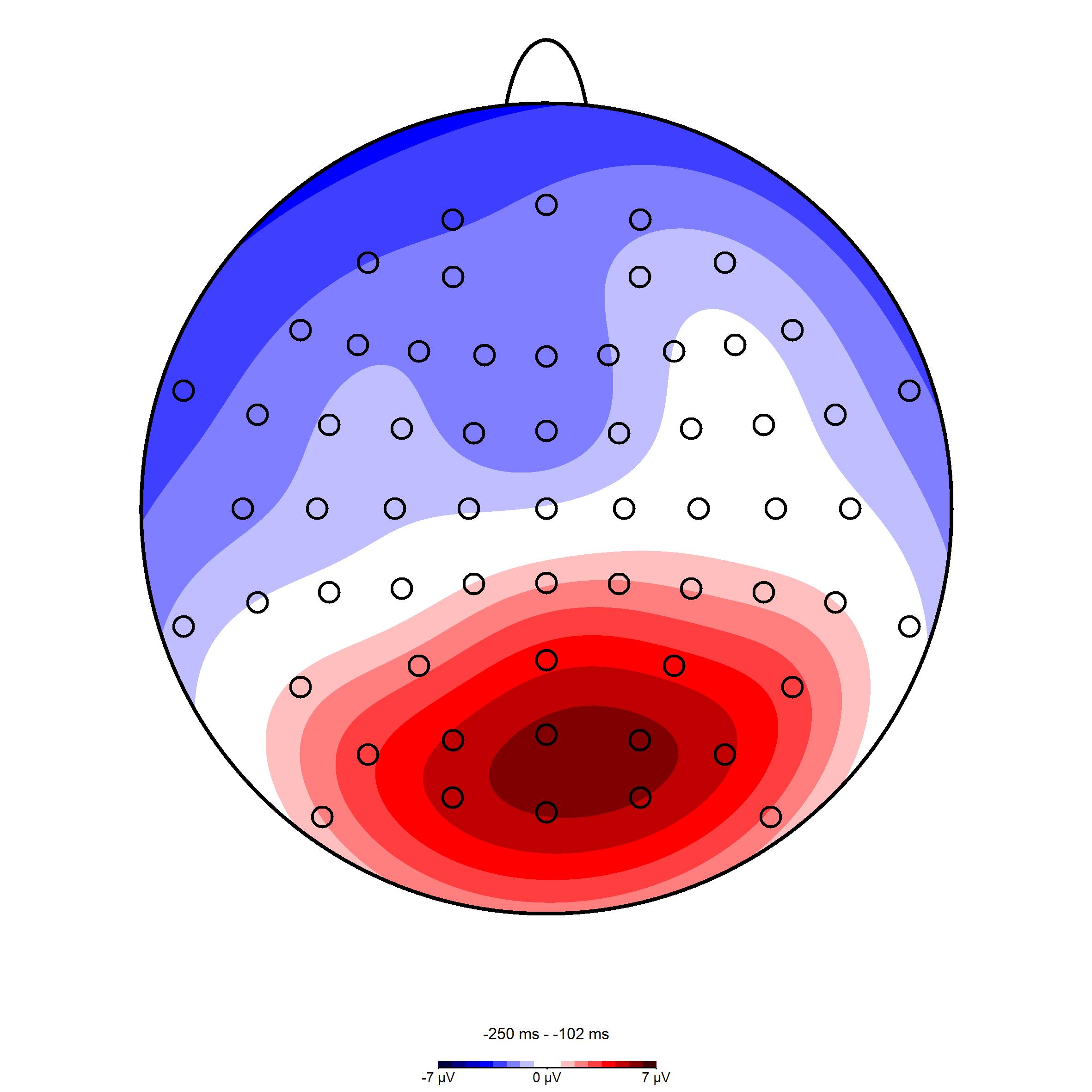 |
| --- | --- | --- |


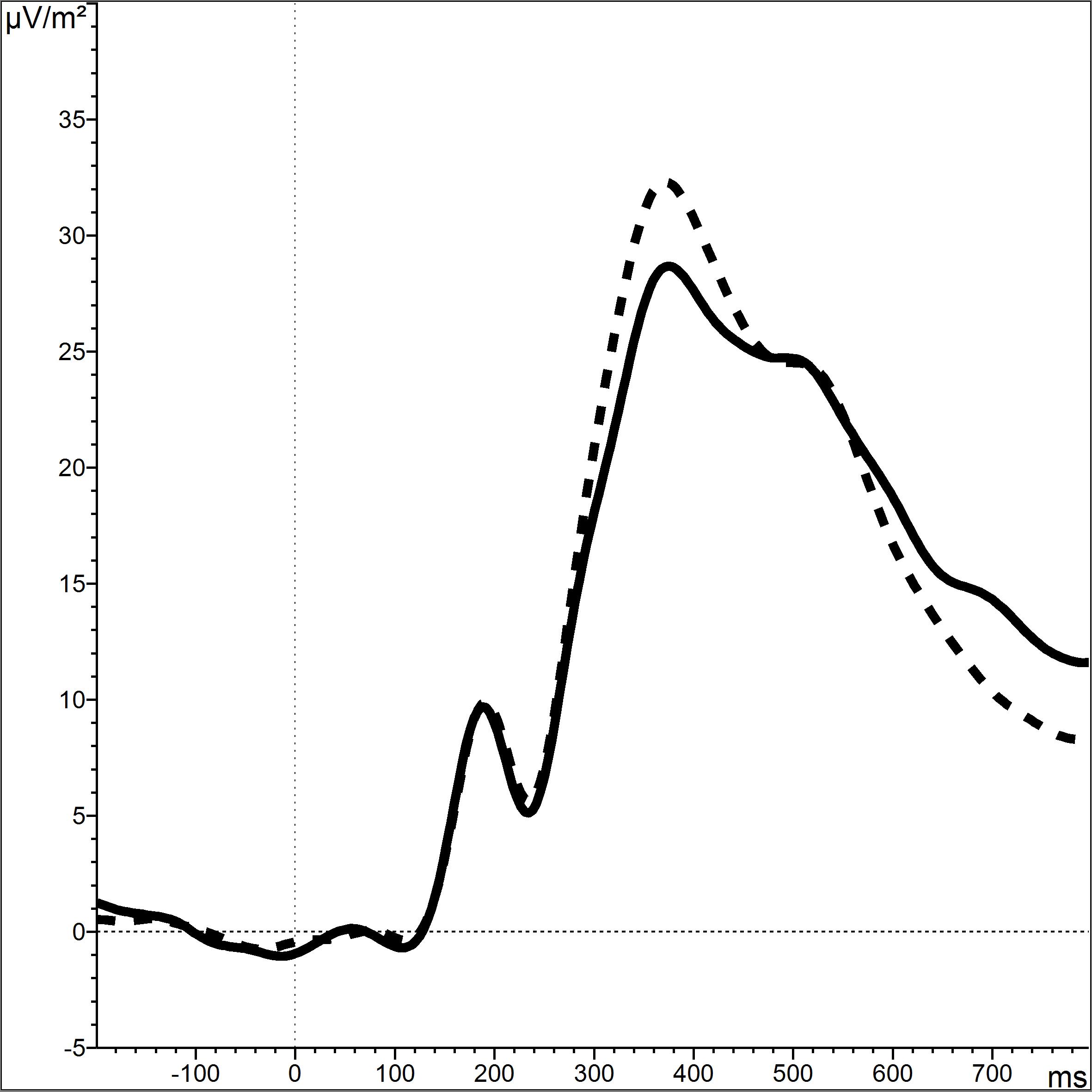

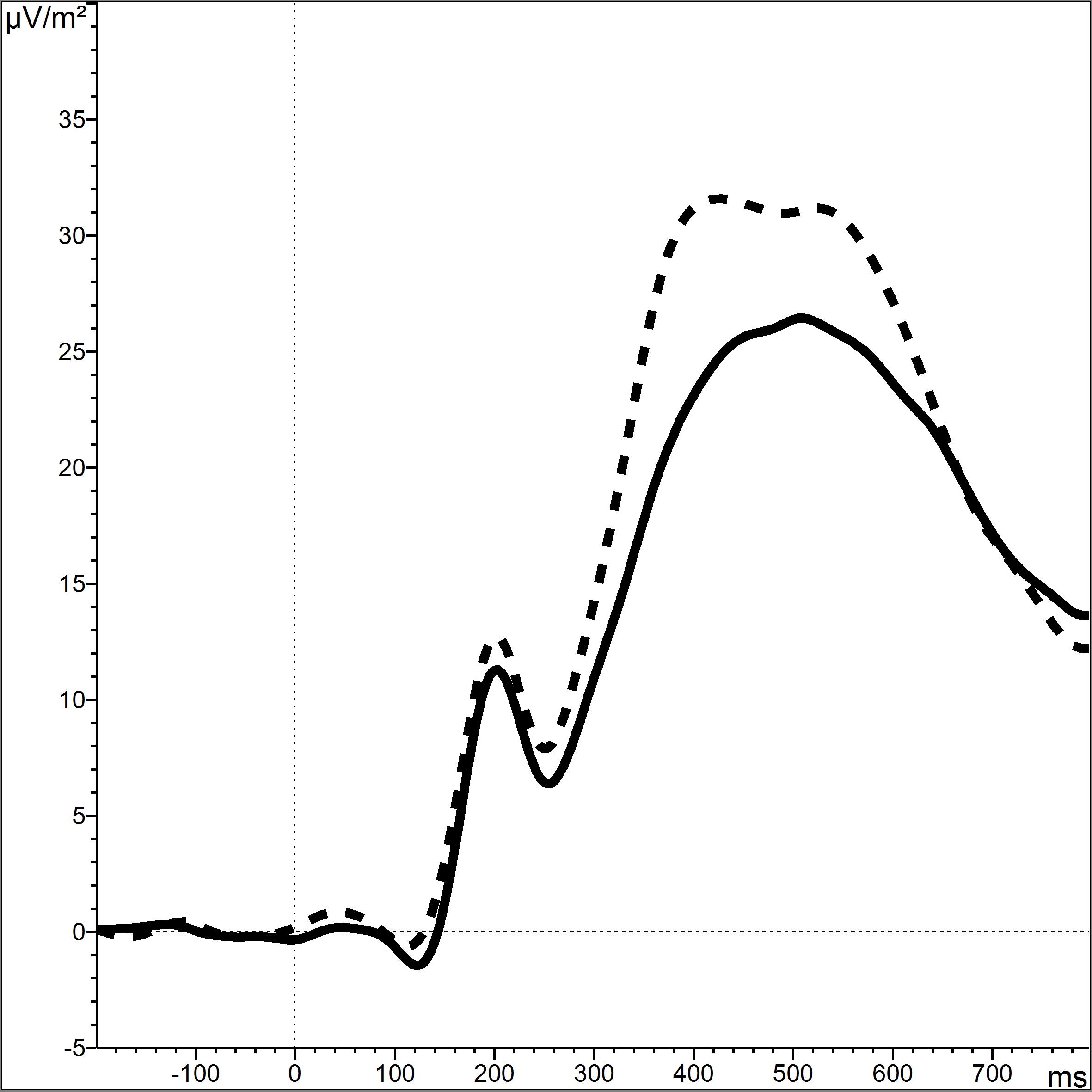


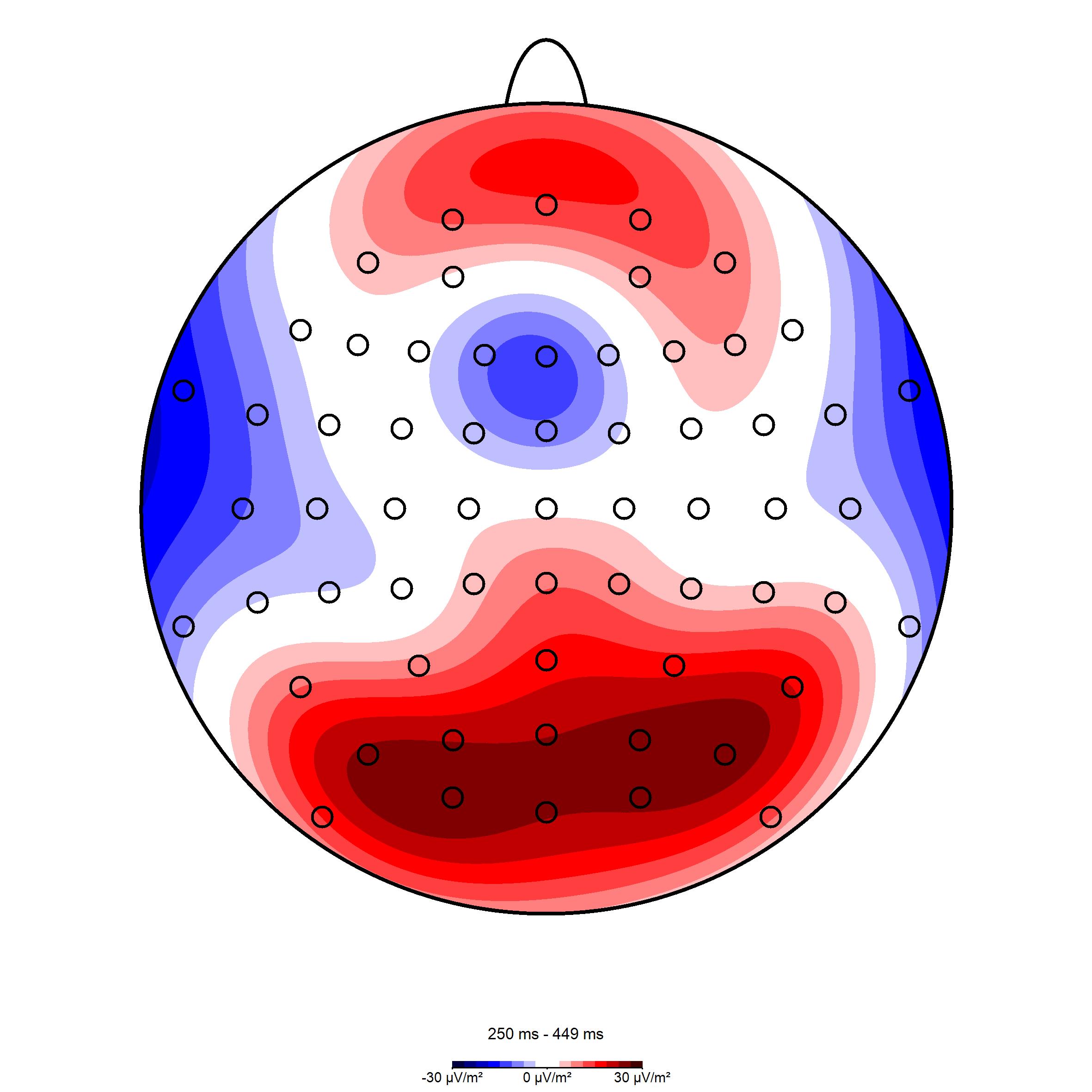

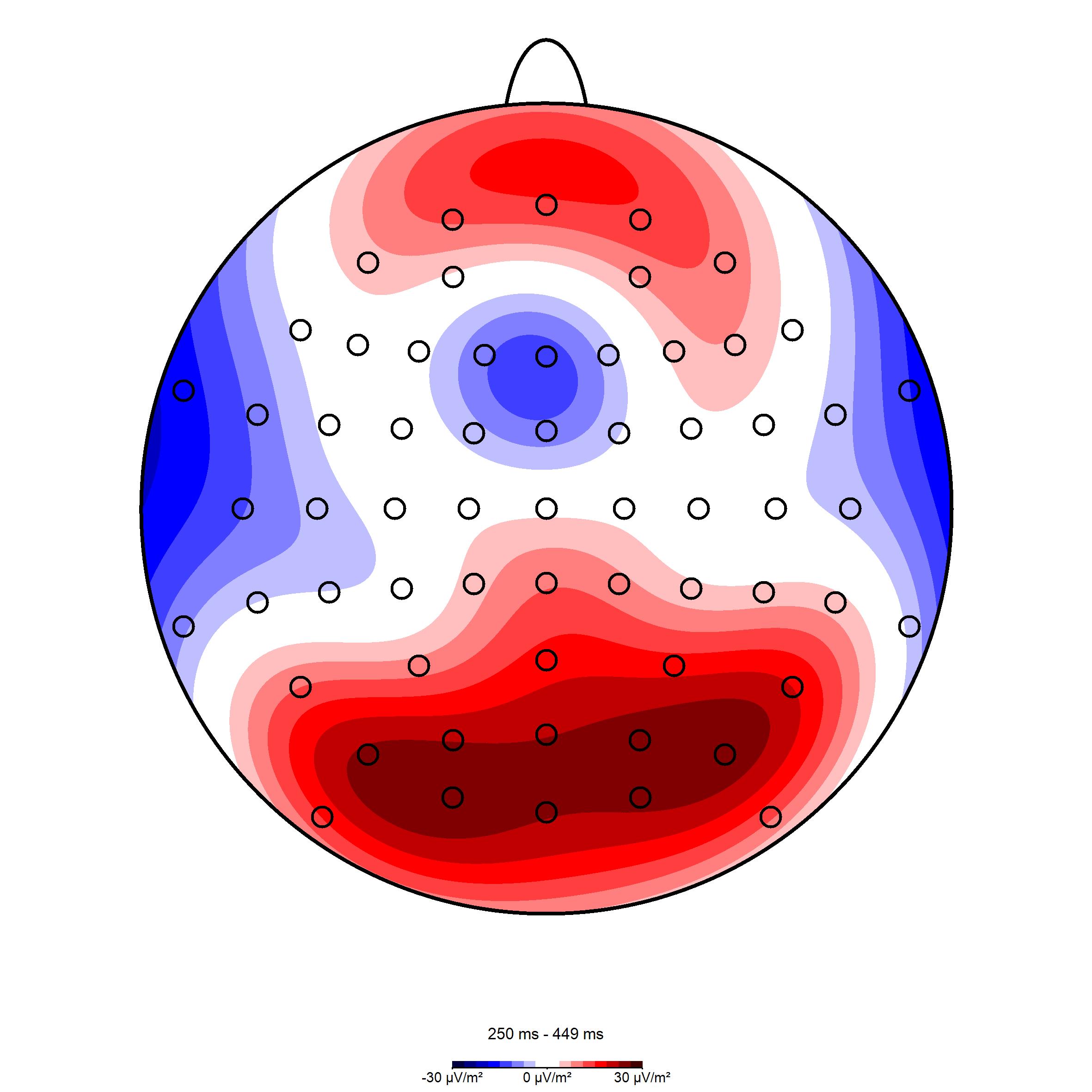

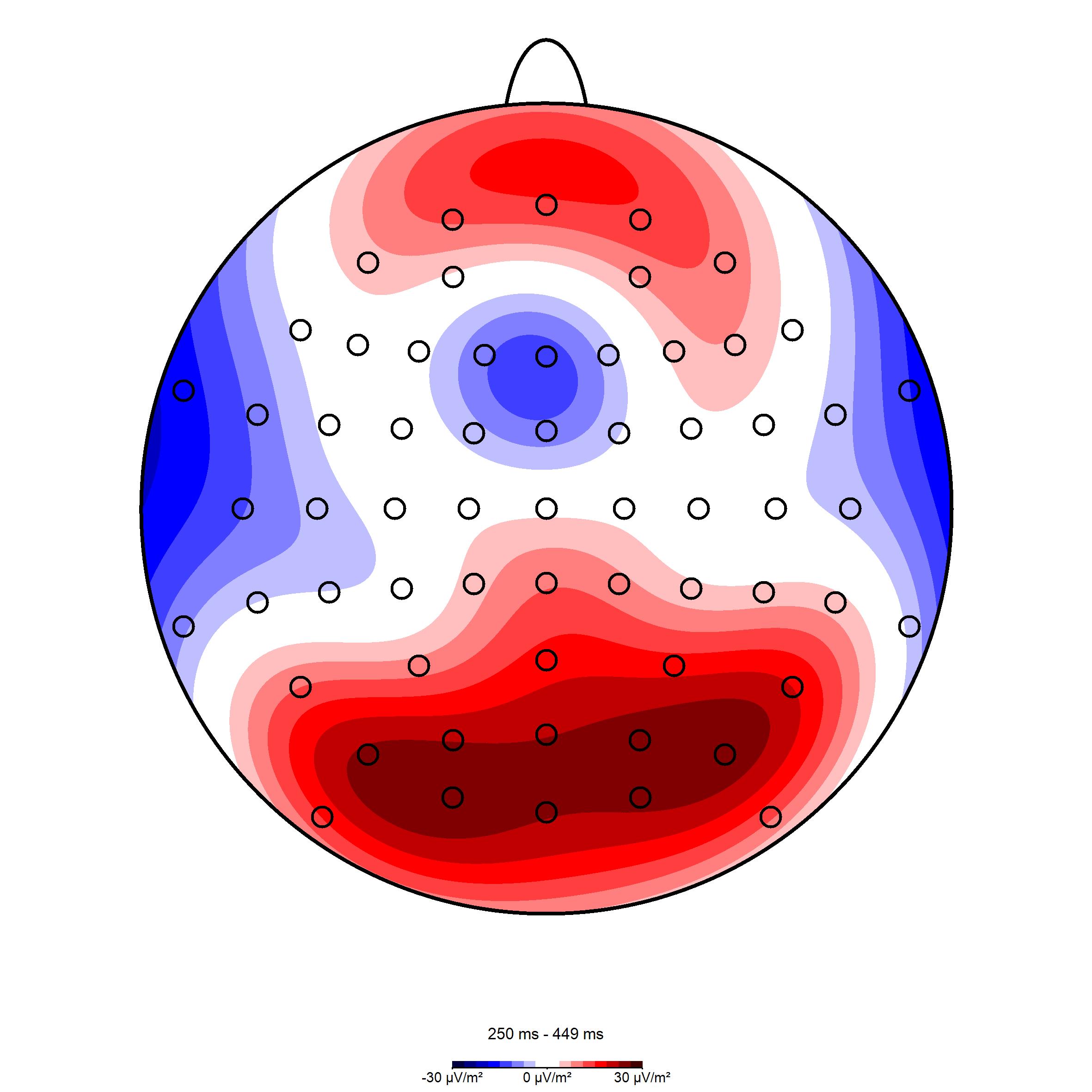

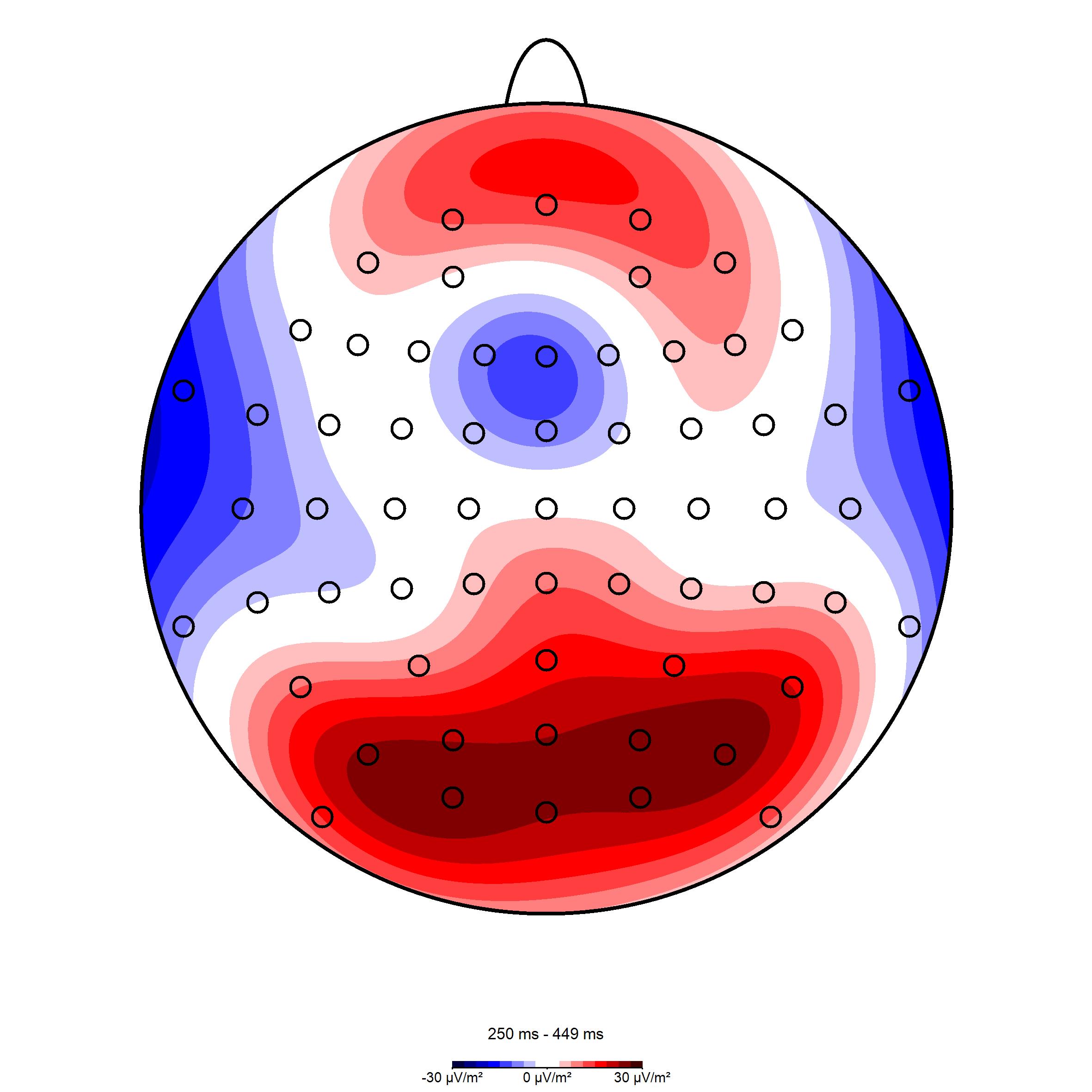


**ADHD Control**

| 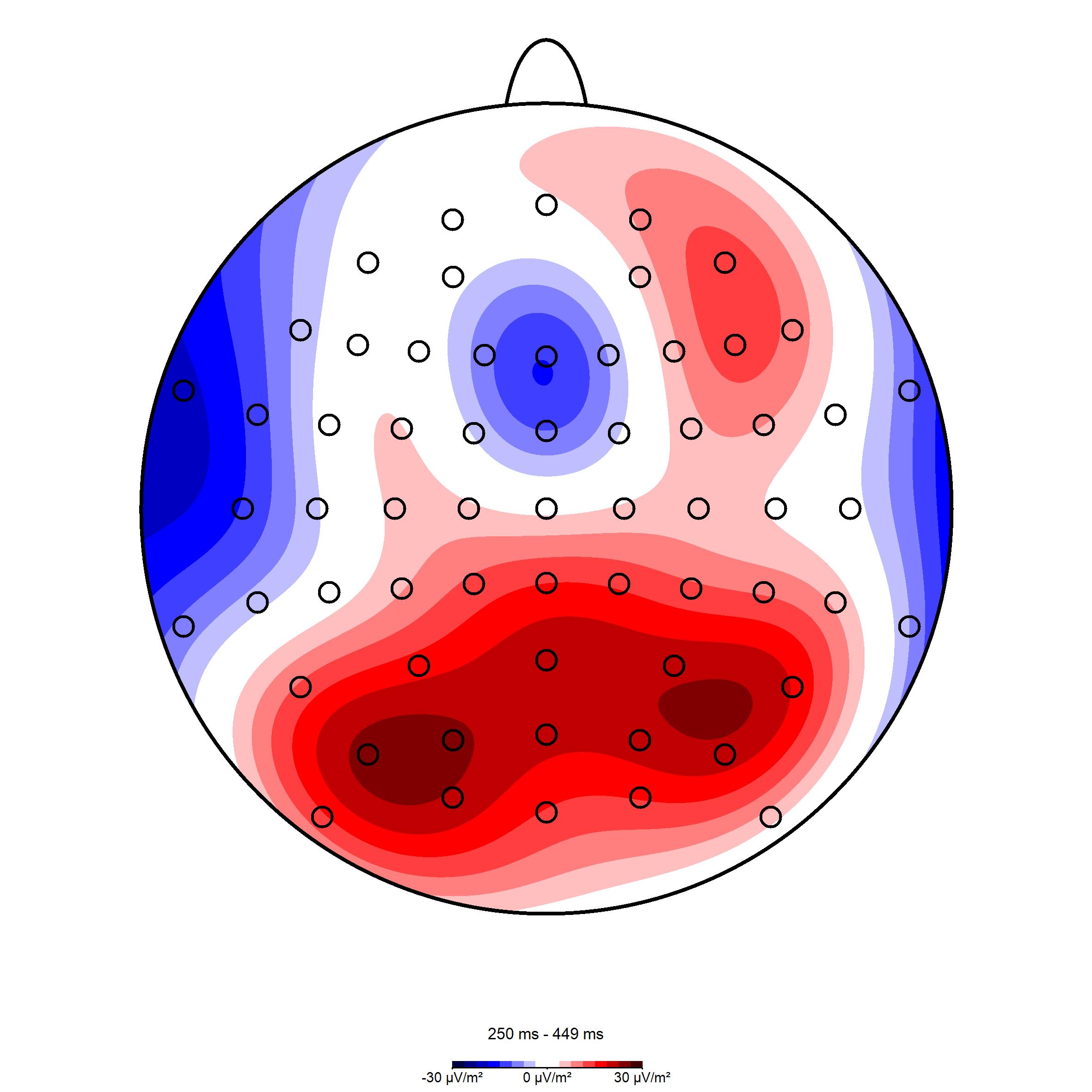 | 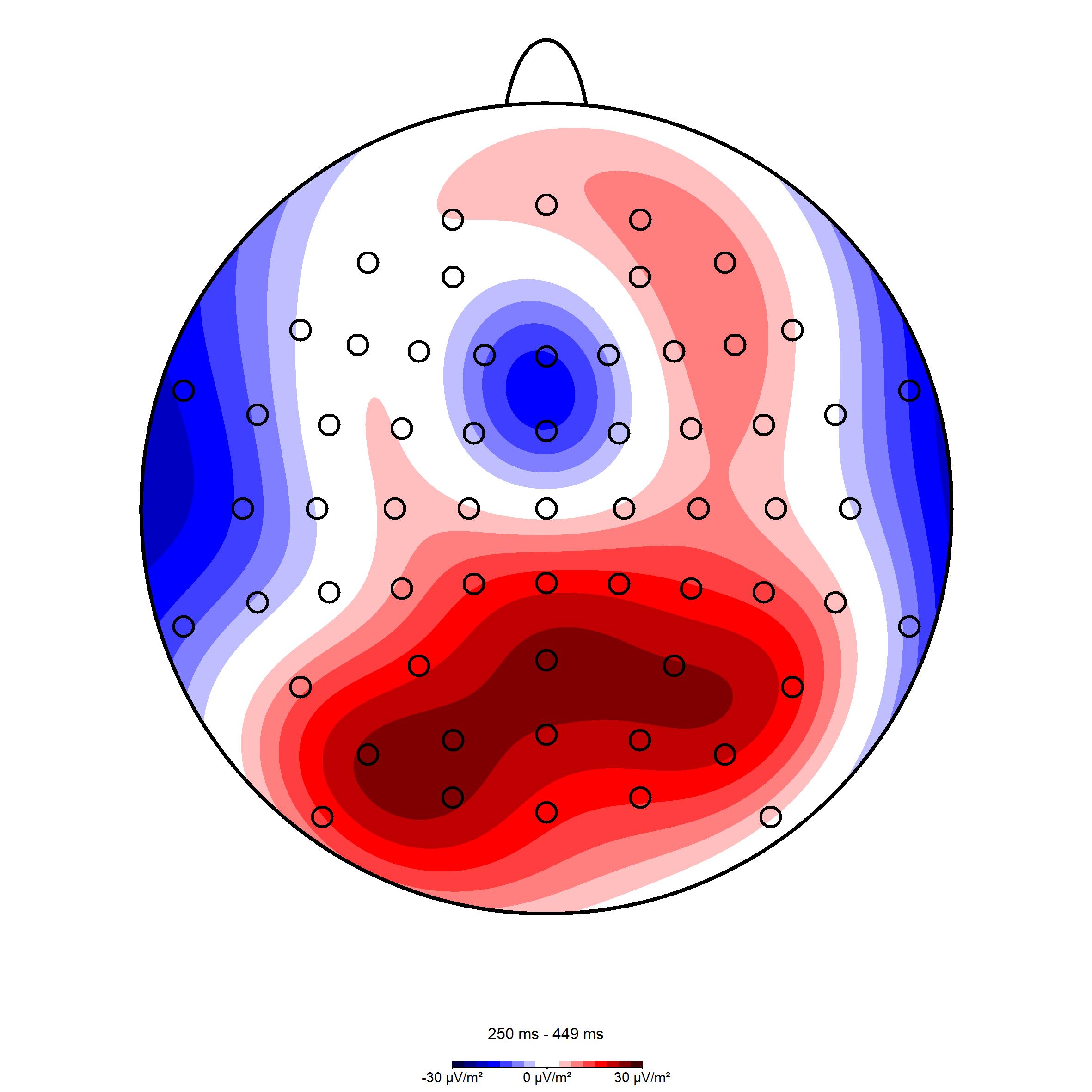 |  |
| --- | --- | --- |

Fast-incentive

| 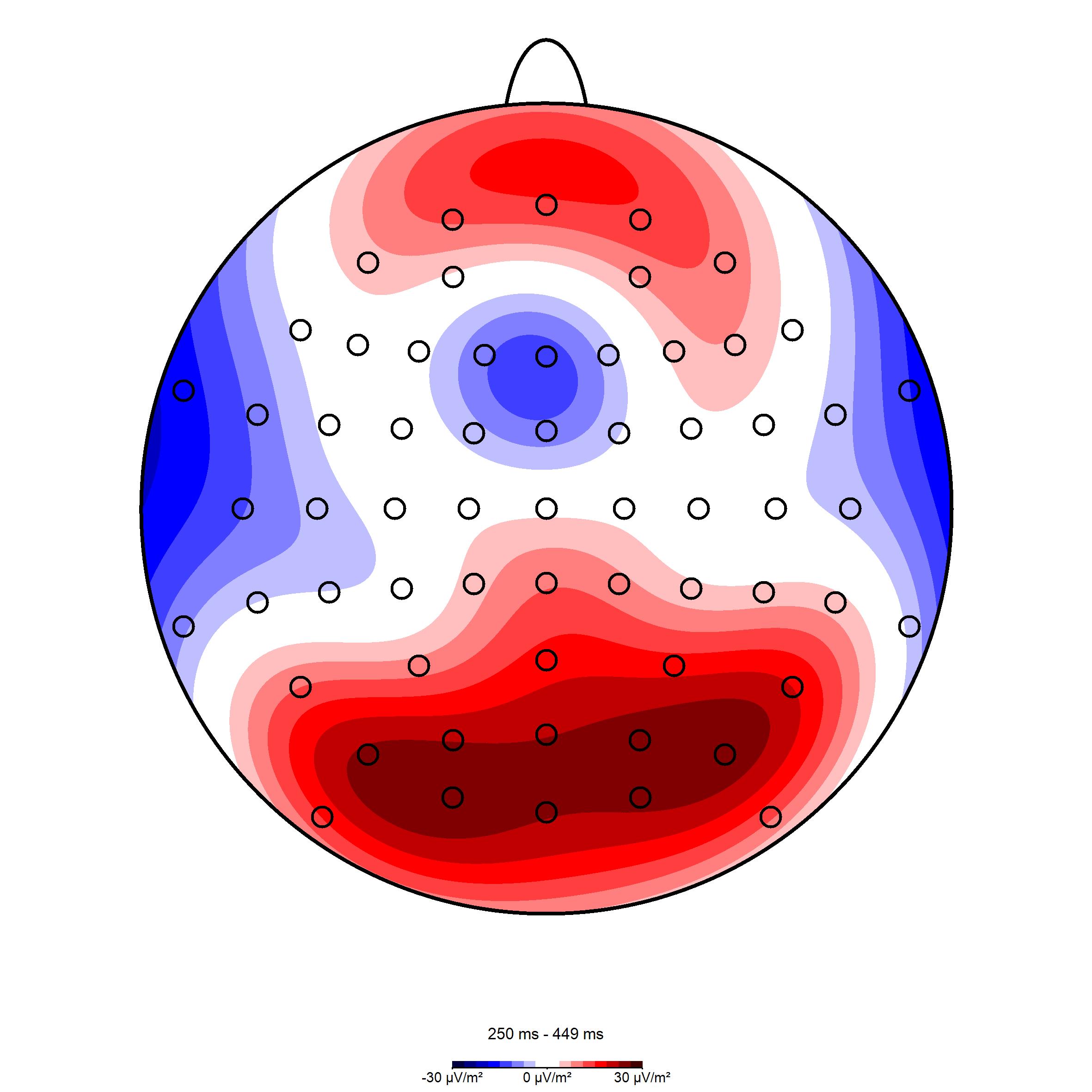 | 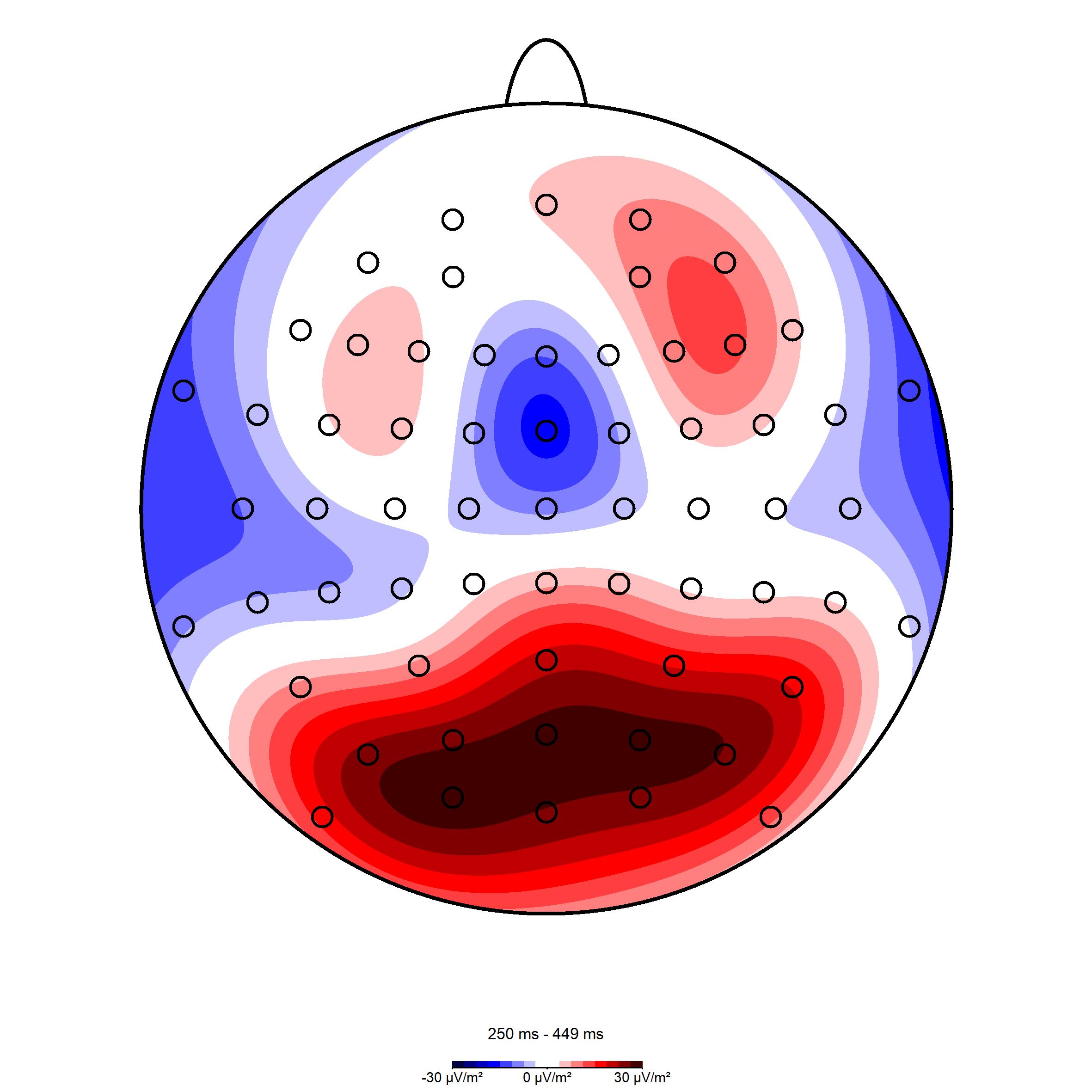 |  |
| --- | --- | --- |

**ADHD Control**

Baseline

**Figure S4.** Topographical and t-maps of the stimulus aligned P3 (250-450ms) at Pz, with technical zero baseline (without baseline subtraction/correction) in the ADHD (solid line) and control (dotted line) groups in the baseline (a) and fast-incentive conditions (b). Significant effects emerged for condition (*z*=6.02, *p*<0.01), group (*z*=-2.23, *p*=0.03), and the group by condition interaction (*z*=3.03, *p*=0.02).

**b) Fast-incentive condition**

**a) Baseline condition**

**
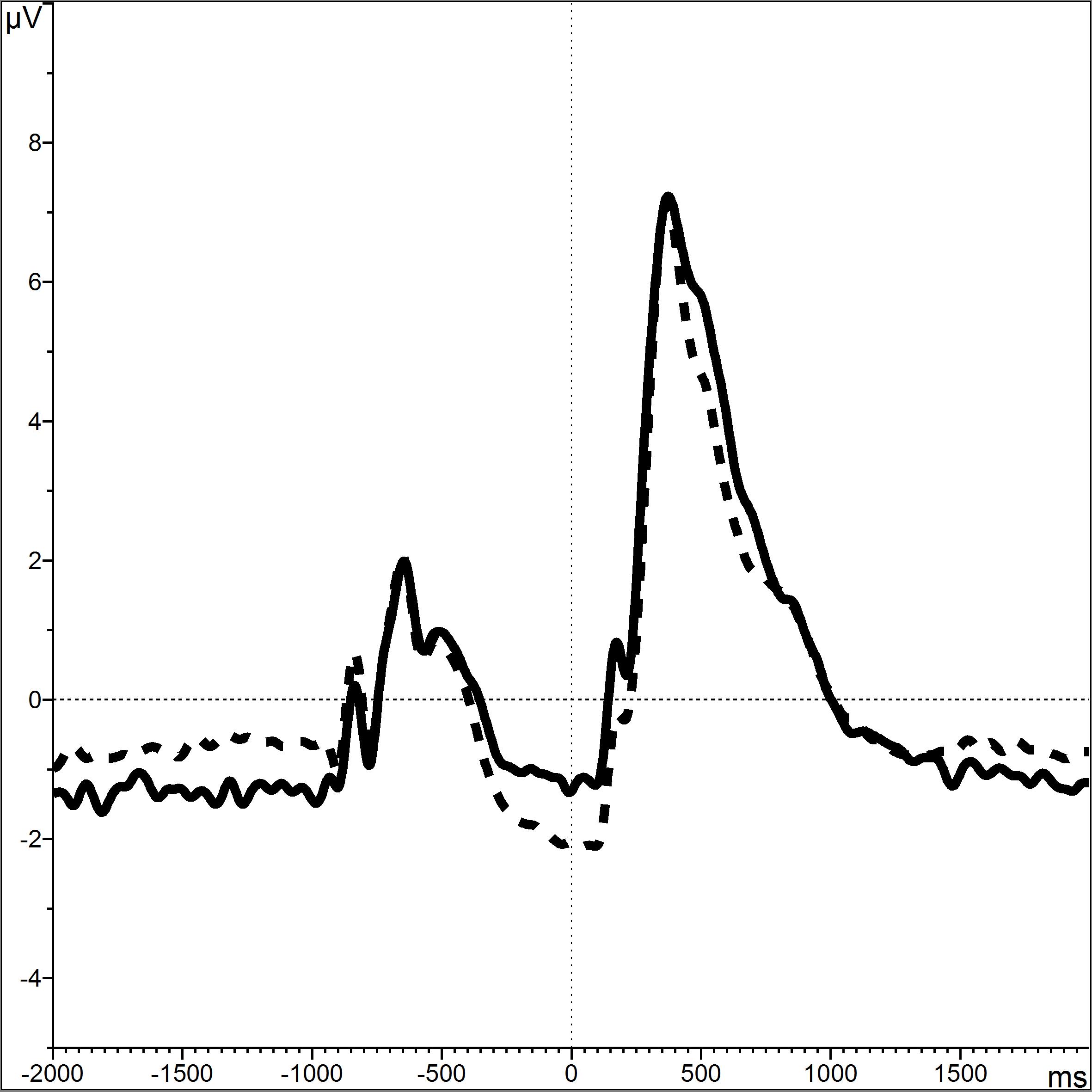

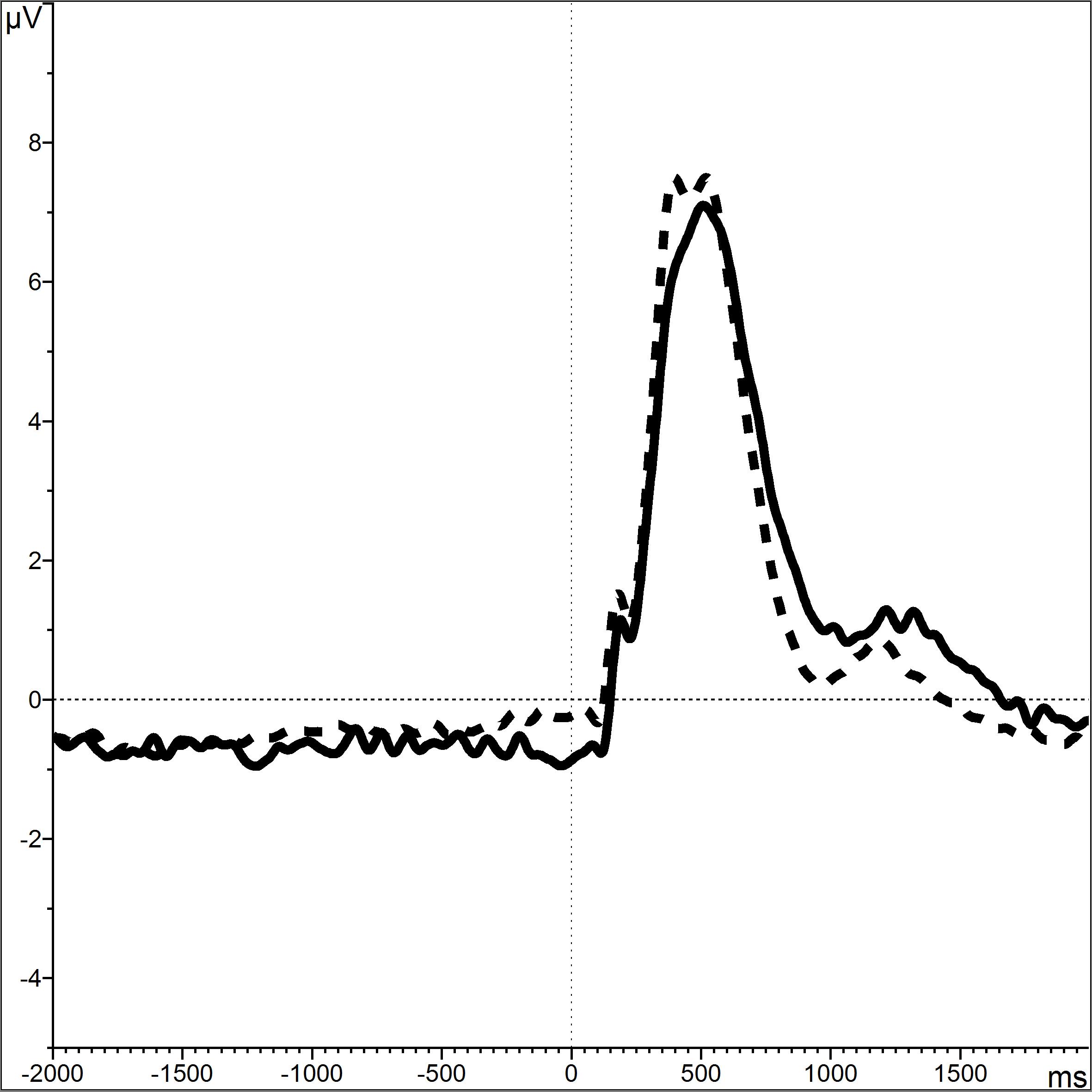
**


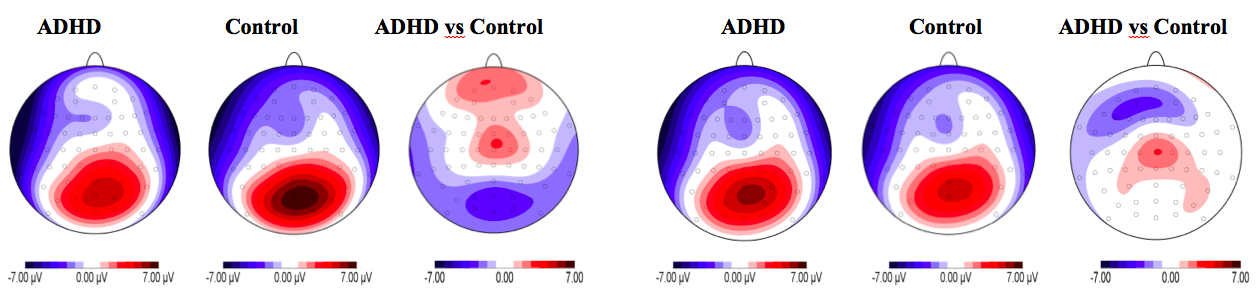

Supplement: Supplementary file 1 — Supplementary material 1 (DOCX 3961 KB) [file 10548_2017_554_MOESM1_ESM.docx]
